# Highly conserved, non-human-like, and cross-reactive SARS-CoV-2 T cell epitopes for COVID-19 vaccine design and validation

**DOI:** 10.1038/s41541-021-00331-6

**Published:** 2021-05-13

**Authors:** Lauren M. Meyers, Andres H. Gutiérrez, Christine M. Boyle, Frances Terry, Bethany G. McGonnigal, Andres Salazar, Michael F. Princiotta, Wiliam D. Martin, Anne S. De Groot, Leonard Moise

**Affiliations:** 1 EpiVax, Inc., Providence, RI USA; 2 Oncovir, Inc., Washington, DC USA; 3 EpiVax Therapeutics, Inc., Providence, RI USA; 4 Center for Vaccines and Immunology, University of Georgia, Athens, GA USA; 5 Institute for Immunology and Immunoinformatics, University of Rhode Island, Providence, RI USA

**Keywords:** Peptide vaccines, Viral infection

## Abstract

Natural and vaccine-induced SARS-CoV-2 immunity in humans has been described but correlates of protection are not yet defined. T cells support the SARS-CoV-2 antibody response, clear virus-infected cells, and may be required to block transmission. In this study, we identified peptide epitopes associated with SARS-CoV-2 T-cell immunity. Using immunoinformatic methods, T-cell epitopes from spike, membrane, and envelope were selected for maximal HLA-binding potential, coverage of HLA diversity, coverage of circulating virus, and minimal potential cross-reactivity with self. Direct restimulation of PBMCs collected from SARS-CoV-2 convalescents confirmed 66% of predicted epitopes, whereas only 9% were confirmed in naive individuals. However, following a brief period of epitope-specific T-cell expansion, both cohorts demonstrated robust T-cell responses to 97% of epitopes. HLA-DR3 transgenic mouse immunization with peptides co-formulated with poly-ICLC generated a potent Th1-skewed, epitope-specific memory response, alleviating safety concerns of enhanced respiratory disease associated with Th2 induction. Taken together, these epitopes may be used to improve our understanding of natural and vaccine-induced immunity, and to facilitate the development of T-cell-targeted vaccines that harness pre-existing SARS-CoV-2 immunity.

## Introduction

Global spread of severe acute respiratory syndrome coronavirus 2 (SARS-CoV-2) has resulted in over 145 million cases of coronavirus disease 2019 (COVID-19), 3.1 million deaths, and global economic disruption in <15 months since the first cases were reported in Wuhan, China. Recovery from natural infection in non-severe disease and resistance to severe disease in younger individuals, despite transmission found in asymptomatic infection, suggest that the immune system can be harnessed to help bring an end to the COVID-19 pandemic by vaccination strategies that recapitulate protective immune responses.

Although immune correlates of COVID-19 protection are not yet defined, several studies show cellular adaptive immune mechanisms contribute to SARS-CoV-2 control (reviewed in ref. ^1^). Humoral immune responses also contribute to protection and have been the focus of current vaccine development efforts. Virus-specific IgM and IgG antibodies are found in nearly all infections^2^. Seroconversion is observed 7–14 days after the onset of symptoms and persists for weeks after virus clearance. Antibody levels wane 4–5 months after infection^3,4^, but durable memory B-cell immunity has been described in mild and severe disease^5^. Antibodies are found against the surface spike glycoprotein and the internal nucleocapsid protein^6^. Neutralizing antibodies target the receptor-binding domain and the N-terminal domain of spike, preventing cell entry via the angiotensin-converting enzyme 2 (ACE2) host receptor^7,8^. Neutralizing antibodies are found in >90% of persons who seroconvert^9^.

Although binding and neutralizing antibody titers track with protection from infection, the contribution of T cells to this protective antibody response and to protection against severe disease may be underestimated. Correlations between a wide range of T-cell responses and protection from infection have begun to emerge. A large prospective study showed numbers of SARS-CoV-2-specific T cells inversely correlate with disease risk^10^. Individuals with low T-cell responses to spike, membrane, and nucleocapsid proteins develop COVID-19, whereas high responders do not, even if seronegative. T-cell breadth is another key feature of the protective response, as patients with mild disease have higher T-cell receptor (TCR) clonality in the blood and bronchoalveolar lavage, in comparison to patients with severe disease^11,12^. T-cell phenotype and function may also help to predict mild vs. severe cases and may be related to protection. Poor outcomes are associated with multiple signs of T-cell impairment including enhanced expression of PD-1 and TIM-3 exhaustion markers^13^, higher inhibitory molecule levels including CTLA-4 and TIGIT^14^, and low frequencies of polyfunctional CD4^+^ and CD8^+^ T cells, as well as CD8^+^ T cells expressing increased levels of GzmA and GzmB in the bronchoalveolar fluid^12,15^. In contrast, non-severe patients present with lower levels of inhibitory molecules and lower GzmB and perforin^14,15^.

SARS-CoV-2 T-cell responses are also linked to protective humoral immunity as spike-specific follicular helper CD4^+^ T-cell (Tfh) frequencies correlate with neutralizing antibody responses^16^. Moreover, in recovered patients, Tfh are found in the periphery at the time of viral clearance and persist into convalescence^17^ in contrast with an absence of lymph node Tfh found in patients who died of COVID-19^18^. In a prospective study of exposed healthcare workers, anti-COVID-19 IgG titers were associated with protection from subsequent PCR test positivity^19^, suggesting that either antibodies or T-cell response, or both, were correlates of protection from subsequent infection. These findings underscore the importance of defining T-cell epitope specificities to better understand COVID-19 immunity and to develop antibody- and T-cell-directed vaccines that exploit T-cell immunity.

Here we use iVAX, a computational epitope identification and vaccine design platform^20^, to identify CD4^+^ and CD8^+^ T-cell epitopes in surface SARS-CoV-2 antigens and analyze their cross-conservation with high- and low-pathogenicity human coronaviruses. We evaluate a subset of epitopes that are predicted to stimulate effector T cells for immune recall using peripheral blood mononuclear cells (PBMCs) from SARS-CoV-2 convalescent and healthy donors. Finally, we immunize human leukocyte antigen (HLA) transgenic mice with effector T-cell epitope peptides to establish a de novo immune response and assess the type 1/type 2 T-cell balance that is induced.

## Results

### In silico prediction of SARS-CoV-2 T-cell targets

To identify SARS-CoV-2 sequences recognized by T cells capable of inducing protective responses in natural infection, we analyzed the T-cell immunogenicity potential of the SARS-CoV-2 surface spike, membrane, and envelope antigens using immunoinformatic tools. As a group, these antigens are structural proteins, potential antibody targets, and are estimated to be produced at higher abundance than other antigens in infected cells^21^. Initially, we predicted CD4^+^ T-cell immunogenicity potential using the EpiMatrix T-cell epitope mapping algorithm and the Wuhan-Hu-1 strain as a reference sequence. Predictions were made using nine HLA class II and six HLA class I supertype alleles representing >95% of the human population^22,23^. For each antigen, we identified regions of high HLA class II epitope density, called clusters, across multiple supertype alleles. A total of 52 epitope clusters containing between 6 and 60 binding motifs each were identified (Table 1). These epitope clusters contain more than 100 total individual binding motifs for each of the 9 supertype alleles, ranging from 109 for DRB1*0301 to 180 for DRB1*0901.

**Table 1:** Immunogenicity potential and human and coronavirus cross-conservation features of SARS-CoV-2 surface antigen epitope clusters.

Antigen	Cluster address	Cluster sequence	EpiMatrix hits Class I^a^	EpiMatrix hits Class II^b^	EpiMatrix cluster Score^c^	Janus Human Homology Score^d^	IEDB coronavirus^e^	SARS-CoV-2 conservation^f^
ENVELOPE	1–25	MYSFVSEETGTLIVNSVLLFLAFVV	25	23	36.95	3.03	1	99.38%
ENVELOPE	14–44	VNSVLLFLAFVVFLLVTLAILTALRLCAYCC	44	60	110.24	3.78	0	99.85%
ENVELOPE	46–75	IVNVSLVKPSFYVYSRVKNLNSSRVPDLLV	20	37	63.92	1.15	0	99.29%
MEMBRANE	13–31	LKKLLEQWNLVIGFLFLTW	22	8	13.01	9.00	1	99.58%
MEMBRANE	25–48	GFLFLTWICLLQFAYANRNRFLYI	28	18	28.42	16.00	0	99.80%
MEMBRANE	42–66	RNRFLYIIKLIFLWLLWPVTLACFV	41	24	39.94	22.00	3	99.80%
MEMBRANE	57–76	LWPVTLACFVLAAVYRINWI	13	12	20.32	9.00	1	99.53%
MEMBRANE	87–114	LVGLMWLSYFIASFRLFARTRSMWSFNP	37	34	58.30	16.00	0	99.86%
MEMBRANE	109–129	MWSFNPETNILLNVPLHGTIL	10	15	26.00	11.00	0	99.83%
MEMBRANE	140–160	IGAVILRGHLRIAGHHLGRCD	4	12	18.30	21.00	0	99.87%
MEMBRANE	165–181	PKEITVATSRTLSYYKL	13	7	11.79	1.00	2	99.51%
MEMBRANE	175–192	TLSYYKLGASQRVAGDSG	4	11	19.56	8.00	2	99.84%
MEMBRANE	196–217	YSRYRIGNYKLNTDHSSSSDNI	13	11	15.31	10.0	0	99.78%
SPIKE	1–26	MFVFLVLLPLVSSQCVNLTTRTQLPP	13	41	71.61	3.19	0	98.07%
SPIKE	25–39	PPAYTNSFTRGVYYP	10	6	12.80	0.17	0	99.63%
SPIKE	39–59	PDKVFRSSVLHSTQDLFLPFF	14	15	23.82	1.94	0	99.11%
SPIKE	52–69	QDLFLPFFSNVTWFHAIH	18	9	16.27	0.22	1	99.03%
SPIKE	61–79	NVTWFHAIHVSGTNGTKRF	7	12	21.41	0.54	0	99.38%
SPIKE	87–103	NDGVYFASTEKSNIIRG	6	7	10.83	0.29	0	99.68%
SPIKE	113–131	KTQSLLIVNNATNVVIKVC	5	23	40.90	0.52	0	99.93%
SPIKE	137–156	NDPFLGVYYHKNNKSWMESE	8	14	23.78	0.00	0	98.89%
SPIKE	154–171	ESEFRVYSSANNCTFEYV	17	11	18.25	0.09	0	99.90%
SPIKE	197–215	IDGYFKIYSKHTPINLVRD	10	12	22.25	0.21	0	99.80%
SPIKE	207–223	HTPINLVRDLPQGFSAL	3	8	14.08	4.25	0	99.39%
SPIKE	232–255	GINITRFQTLLALHRSYLTPGDSS	16	30	55.01	1.03	0	98.78%
SPIKE	266–288	YVGYLQPRTFLLKYNENGTITDA	12	16	23.08	0.84	0	99.92%
SPIKE	309–329	EKGIYQTSNFRVQPTESIVRF	7	10	13.16	0.00	1	99.73%
SPIKE	335–353	LCPFGEVFNATRFASVYAW	7	8	10.23	0.38	0	99.90%
SPIKE	347–364	FASVYAWNRKRISNCVAD	5	7	11.13	0.63	0	99.97%
SPIKE	362–380	VADYSVLYNSASFSTFKCY	19	20	36.57	0.95	0	99.95%
SPIKE	442–468	DSKVGGNYNYLYRLFRKSNLKPFERDI	20	33	58.42	1.03	0	99.82%
SPIKE	483–500	VEGFNCYFPLQSYGFQPT	10	11	17.96	0.00	0	99.59%
SPIKE	492–508	LQSYGFQPTNGVGYQPY	3	7	10.08	0.14	0	99.69%
SPIKE	508–529	YRVVVLSFELLHAPATVCGPKK	12	15	24.27	3.35	3	99.57%
SPIKE	536–554	NKCVNFNFNGLTGTGVLTE	2	12	18.82	0.00	2	99.88%
SPIKE	715–729	PTNFTISVTTEILPV	8	6	12.63	0.00	1	99.91%
SPIKE	749–774	CSNLLLQYGSFCTQLNRALTGIAVEQ	13	21	30.52	0.64	1	99.88%
SPIKE	797–816	FGGFNFSQILPDPSKPSKRS	4	8	10.25	0.13	1	99.75%
SPIKE	818–837	IEDLLFNKVTLADAGFIKQY	7	12	18.64	1.69	2	99.78%
SPIKE	852–869	AQKFNGLTVLPPLLTDEM	4	10	15.82	1.64	3	99.80%
SPIKE	866–884	TDEMIAQYTSALLAGTITS	11	13	21.49	1.00	0	99.91%
SPIKE	895–911	QIPFAMQMAYRFNGIGV	15	7	10.20	0.00	3	99.91%
SPIKE	919–945	NQKLIANQFNSAIGKIQDSLSSTASAL	12	18	24.88	1.35	0	99.24%
SPIKE	934–949	IQDSLSSTASALGKLQ	6	7	11.21	3.11	1	99.63%
SPIKE	956–981	AQALNTLVKQLSSNFGAISSVLNDIL	14	30	52.49	1.50	2	99.94%
SPIKE	990–1012	EVQIDRLITGRLQSLQTYVTQQL	8	12	16.19	0.29	2	99.97%
SPIKE	1010–1029	QQLIRAAEIRASANLAATKM	7	14	25.75	2.73	2	99.95%
SPIKE	1044–1063	GKGYHLMSFPQSAPHGVVFL	9	8	11.27	0.50	3	99.90%
SPIKE	1126–1141	CDVVIGIVNNTVYDPL	5	9	16.52	0.44	3	99.89%
SPIKE	1152–1167	LDKYFKNHTSPDVDLG	3	7	12.63	0.00	2	99.81%
SPIKE	1166–1183	LGDISGINASVVNIQKEI	5	9	15.12	1.00	2	99.73%
SPIKE	1214–1231	WYIWLGFIAGLIAIVMVT	13	11	18.34	2.42	2	99.58%

^a^Number of class I epitope 9-mers and 10-mers identified for potential binding to six supertype alleles.

^b^Number of class II epitope 9-mers identified for potential binding to nine supertype alleles.

^c^T-cell immunogenicity potential considering deviation in predicted T-cell epitope content from random expectation. T-cell epitope clusters scoring above 10 are considered potentially immunogenic.

^d^Average depth of coverage within the human proteome for the HLA-binding peptides contained within the T-cell epitope cluster.

^e^Number of coronavirus T-cell epitopes in IEDB with >80% similarity to T-cell epitope clusters.

^f^Percentage of strains isolated between January and December 2020 (*n* = 16,450) that contain identical epitope clusters.

The CD8^+^ T-cell immunogenicity potential of these clusters was evaluated and showed that multiple putative HLA class I epitopes overlap in regions of high HLA class II epitope density (Table 1). These epitope clusters are therefore expected to recall both CD4^+^ and CD8^+^ T-cell responses in individuals with SARS-CoV-2 history, and may stimulate both CD4^+^ and CD8^+^ T-cell immunity in a T-cell-directed vaccine. We also investigated the conservation of the Wuhan-Hu-1 clusters in other SARS-CoV-2 isolates and determined that they are identical to clusters found in >98.45% of strains isolated between January 2020 and January 2021 (Table 1). Highly conserved peptides such as these are useful for vaccines and as reagents for assays that interrogate T-cell responses using samples from natural infection and immunization.

HLA class II ligands may stimulate effector or regulatory CD4^+^ T cells leading to divergent immunological outcomes. HLA-binding predictions do not distinguish between these possibilities. They account for peptide interactions with the HLA-binding groove and overlook potential interactions with the TCR. As the T-cell repertoire is shaped by training on human T-cell epitopes, we routinely assess potential for regulatory T-cell induction by screening the TCR face of epitopes for homology with self-antigens using the JanusMatrix algorithm^24^. For each cluster, the average depth of coverage in the human proteome was calculated holding all TCR-facing positions fixed and allowing HLA-facing positions to vary, while requiring human sequences to bind to the same HLA alleles as the SARS-CoV-2 sequences. JanusMatrix analysis revealed that each SARS-CoV-2 protein contains clusters with significant human homology scores (>2). This was also true for some of the 52 selected clusters. Seventeen (32.7%) were found to have elevated regulatory T-cell induction potential based on high JanusMatrix homology with the human proteome and, in contrast, 35 (67.3%) were considered more likely to induce effector T-cell responses. The results suggest that different CD4^+^ T-cell subsets may be activated by these epitopes in the course of SARS-CoV-2 infection and measurement of recall responses in vitro.

Notably, five spike clusters coincide with mutations in the SARS-CoV-2 B.1.1.7 variant of concern that emerged in the United Kingdom^25^, four in the South Africa B.1.351 variant^26^, three in the US (California) B.1.427 variant^27^, and six in the Brazil P.1 variant (Supplementary Table 1)^28^. The hACE2-contacting N501Y mutation^29^, found in the UK, South Africa, and Brazil variants and shown to increase affinity for hACE2^30^, reduces the immunogenicity potential of cluster 492–508, as the class II HLA EpiMatrix cluster score falls below the significance threshold of 10. In contrast, the E484K mutation in the South Africa and Brazil variants, which escapes neutralizing antibody immunity^31^, results in no change in T-cell immunogenicity potential of cluster 483–500. The majority of mutations across the variants have no significant impact in immunogenicity potential. Only the LLA241–243del mutation in the South Africa variant in cluster 232–255 and the L452R mutation in the US (California) variant in cluster 442–468 reduce the class II HLA EpiMatrix cluster score by >10 points; however, several binding motifs remain in both, indicating their potential to bind HLA persists. Overall, >90% of binding motifs in the spike clusters we identified are preserved in major variants of SARS-CoV-2. JanusMatrix predicted no significant change in homology with self-antigens for all clusters in all variants.

We designed 32 peptides for synthesis by manually editing clusters to center effector epitopes and remove epitopes with significant human homology. We used these 32 peptides to probe T-cell recognition of the putative effector T-cell epitopes. The peptides included 1 envelope sequence, 8 sourced from the membrane, and 23 from the spike (Table 2). Spike peptides comprise 14 in the S1 domain, including 5 receptor binding domain (RBD) sequences, and 9 in the S2 domain.

**Table 2:**
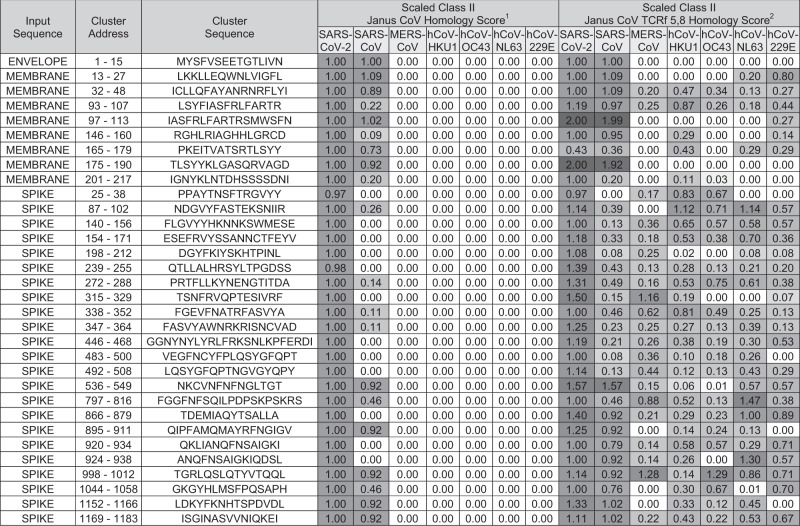
Coronavirus cross-conservation features of effector T-cell epitope peptides screened in immunoassays.

^1^Scaled average depth of coverage within coronavirus data sets requiring identical TCR-facing residues in positions 2, 3, 5, 7, and 8.

^2^Scaled average depth of coverage within coronavirus data sets requiring identical TCR-facing residues in positions 5 and 8.

Janus CoV homology scores were scaled according to the number of sequences in each dataset. Values are shaded from white to dark gray with increasing magnitude.

### SARS-CoV-2 epitope conservation with related coronaviruses at the TCR face

We also investigated the conservation of the selected peptides with related coronaviruses that infect humans, including highly pathogenic SARS-CoV and Middle East respiratory syndrome coronavirus (MERS-CoV), and low-pathogenicity common cold coronaviruses (CCCs) OC43, HKU1, NL63, and 229E. Prior exposure to these viruses may have established T-cell memory that can be recalled upon SARS-CoV-2 infection and vaccination. As well, SARS-CoV-2 infection and vaccination establishes T-cell memory that can influence responses in future infections to these viruses or yet-to-emerge coronaviruses. To identify potentially cross-reactive sequences, we screened the TCR face of SARS-CoV-2 epitopes for conservation with coronaviruses that infect humans, using the JanusMatrix algorithm (Table 2). All of the membrane and envelope sequences shared identical TCR-face patterns with SARS-CoV. Half the spike clusters were unique to SARS-CoV-2 and the other half were conserved with SARS-CoV. No cross-conservation with MERS-CoV nor CCCs was observed. Given reports of pre-existing T-cell immunity in people with no SARS-CoV-2 experience, we relaxed the requirement for 100% identity at every TCR-face position. Fixing two positions shown to be extensively involved in TCR interactions (positions 5 and 8)^32^, JanusMatrix predicted an expanded cross-conservation landscape for SARS-CoV-2 spike and membrane peptides (Table 2). Of the 32 peptides, only 2 are SARS-CoV family specific. Seventeen (53.1%) are cross-conserved across SARS-CoV, MERS-CoV, and all CCCs; 12 (37.5%) are cross-conserved in SARS-CoV, MERS-CoV, and 1–3 of the 4 CCCs. One peptide is cross-conserved in SARS-CoV and CCCs but not MERS-CoV.

### Clinical cohort

Persons with and without SARS-CoV-2 infection history were selected to provide PBMC samples for validation of predicted T-cell epitopes. COVID-19 convalescents with PCR-confirmed SARS-CoV-2 infection (*N* = 15) over March–June 2020 were recruited between 30 and 180 days after their most recent positive test and a minimum of 14 days after symptoms resolved (Table 3 and Supplementary Table 2). Donors exhibited a wide range of COVID-19 symptoms and experienced either mild or moderate disease according to the World Health Organization criteria^33^. Blood draws from convalescents ranged from ~1 to 6 months from diagnosis. Healthy individuals (*N* = 10) provided cell samples from February 2016 up to November 2019 and had no opportunity for SARS-CoV-2 exposure. Both cohorts contain a balanced proportion of females and males, and similar average age and age range. Sixty percent of convalescent donors are from racial and ethnic minorities. No ethnicity information is available for the healthy cohort.

**Table 3:** COVID-19 convalescent and healthy donor cohort characteristics.

	Naive (*N* = 10)	Convalescent (*N* = 15)
Age (years)		
Average	48.5	44.8
Range	26–79	23–71
Gender		
Female	40% (4/10)	53.33% (8/15)
Male	60% (6/10)	46.67% (7/15)
Ethnicity		
Caucasian	NA	40% (6/15)
Hispanic/Latino	NA	33.33% (5/15)
African American	NA	6.67% (1/15)
Asian/Pacific Islander	NA	20% (3/15)
Disease severity		
Mild	NA	73.33% (11/15)
Moderate	NA	26.67% (4/15)
Severe	NA	0
Critical	NA	0
Recruitment		
Sample collection date	Feb 2016–Nov 2019	May–Oct 2020
Date tested positive	NA	Mar–Jun 2020
Time since last positive test (days)	NA	33–158
Time since symptoms resolved (days)	NA	26–116
Date last tested positive	NA	Mar–Jun 2020
Date symptoms resolved	NA	Mar–Jun 2020
PCR positivity	NA	100% (15/15)

### Predicted epitopes are recognized in natural SARS-CoV-2 infection

To determine what predicted effector CD4^+^ T-cell epitope clusters are recognized by T cells elicited by SARS-CoV-2 infection, we conducted ex vivo interferon-γ (IFNγ) Fluorospot assays using whole PBMC preparations from convalescent donors. Immune recall in the Fluorospot assay was stimulated using either individual or pooled peptides. Responses that were both ≥25 spot-forming cells over background and ≥5-fold over background were considered positive. In preliminary studies, a small cohort of donor PBMCs were stimulated with lower concentrations of peptide (0.313 μg/mL per pooled peptide, 10 μg/mL for individual peptide stimulations). These concentrations were increased to potentially heighten assay sensitivity; however, we found that there was no significant difference in the frequency of epitope-specific clones nor in the number of peptides detected per donor between the assay conditions; thus, both data sets are combined here.

**Fig. 1:**
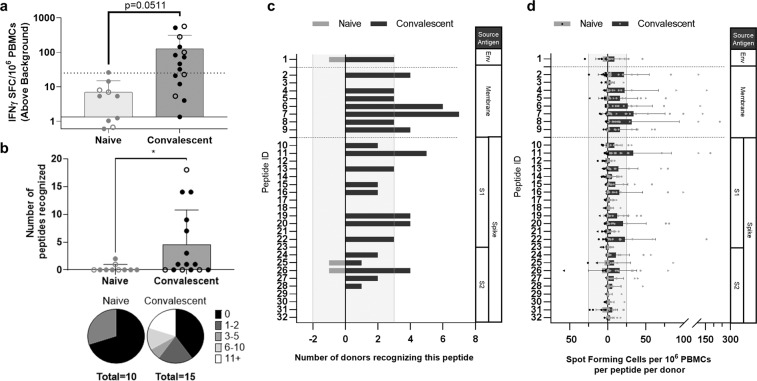
Predicted SARS-CoV-2 T-cell epitopes are antigenic ex vivo in COVID-19 convalescent donors but not healthy donors. **a** Convalescent (*n* = 15) and pre-SARS-CoV-2 pandemic donors (naive, *n* = 10) were stimulated with the total peptide pool consisting of our 32 predicted epitopes and IFNγ-producing cells were measured by Fluorospot assay. Open circles identify responses to low-dose restimulation at 0.313 μg/mL per peptide; closed circles are stimulations at 10 μg/mL per peptide. Dotted horizontal line indicates positivity criteria at SFC/10^6^ splenocytes = 25; solid horizontal line indicates threshold of detection for assay (*t* = 2.058, d.f. = 23). **b** IFNγ responses to individual peptides were also assessed, identifying the breadth of response in individual donors (*t* = 2.113, d.f. = 23), **c** the frequency of responses to unique peptides within the cohort (vertical lines denote 20% of each cohort), **d** and the depth of response indicated by the frequency of IFNγ-producing, epitope-specific cells (vertical lines indicate positivity criteria at SFC/10^6^ splenocytes = 25). Error bars depict SD. *p < 0.05.

Overall, we found that the majority of COVID-19 convalescent donor samples (9/15; 60%) responded to a pool of the 32 peptides (Fig. 1a). In contrast, only a single healthy control donor sample out of ten demonstrated immune recall, just above the threshold, to this pool. Individual peptide stimulations showed that COVID-19 convalescent donor samples had positive responses to envelope, membrane, and spike peptides, including responses in the S1 and S2 domains and RBD (Fig. 1d).

For 21/32 (66%) peptides, a recall response was observed in PBMCs from at least 1 convalescent donor (corresponding to 1/1 envelope-derived epitope, 7/8 (87.5%) membrane-derived epitopes, and 13/23 (56.5%) spike-derived epitopes); 14/32 (44%) peptides were confirmed in at least 20% of convalescent donor samples (Fig. 1c).

The number of peptides confirmed to be recognized per donor sample ranged from 0 to 18 with an average response of 4–5 peptides per convalescent donor (Fig. 1b). Of samples derived from healthy donors, only two recognized any of the peptides ex vivo: the aforementioned donor sample responding to the total peptide pool recalled the sole envelope epitope (peptide 1) and an S2-derived sequence (peptide 25), whereas another donor sample demonstrated reactivity to an S2 domain sequence (peptide 26). Although rare, these findings suggest that there may be a memory response that cross-reacts with prior CCC infection.

**Fig. 2:**
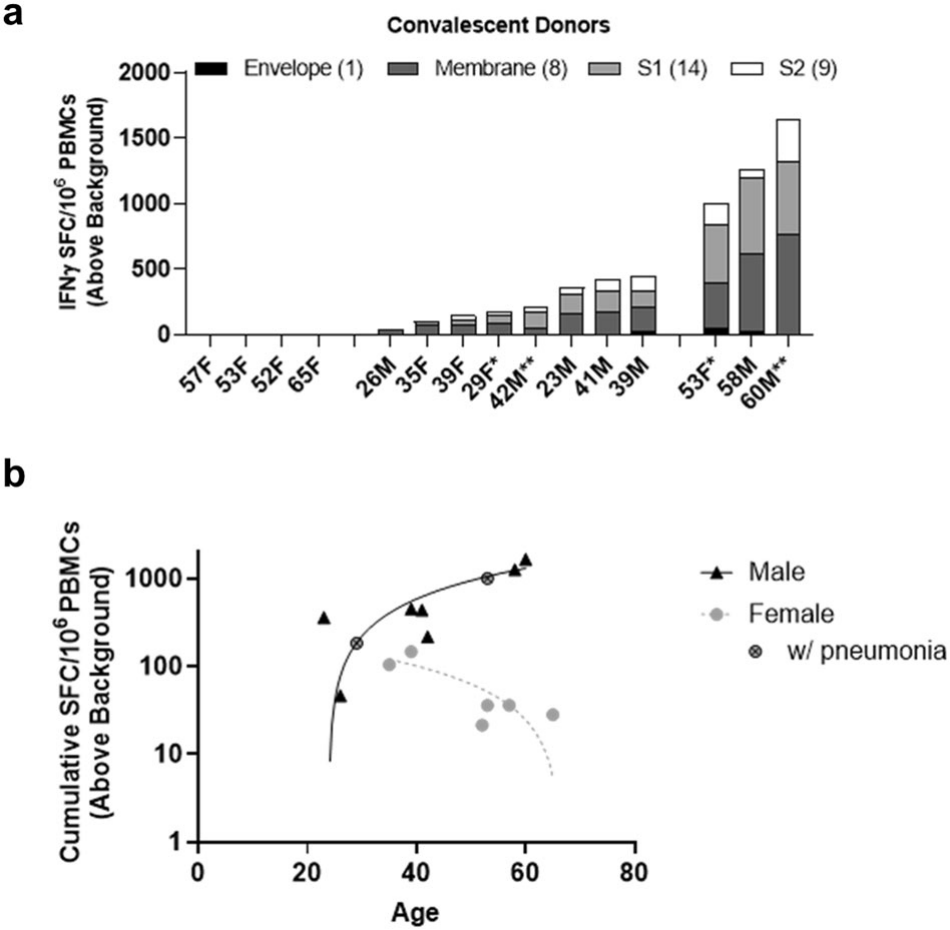
SARS-CoV-2 experienced individuals exhibit variable immune recall responses ex vivo. **a** Significant responses to individual peptides (combined per source antigen) identify three distinct immunotype cohorts within covalescent donors (donor notations: *pneumonia, **hospitalized, non-ICU). **b** Correlation of cumulative T-cell responses and age according to gender. Male cohort (*n* = 7, *R*^2^ = 0.7571, *p* = 0.0109). Female cohort (*n* = 8, *R*^2^ = 0.00001, *p* = 0.9934). Mild subset of female cohort (*n* = 6, *R*^2^ = 0.7034, *p* = 0.0368).

Given the variable patterns of epitope-specific responses, we also evaluated the cumulative response to specific antigens by batching the response to individual peptides in convalescent donors (Fig. 2a). As expected from the individual donor peptide analysis, T cells specific for membrane-derived epitopes were most frequently recalled with 11/15 donor samples demonstrating significant anti-membrane responses and membrane epitope-specific T cells making up >40% of the total recall response in 10/11 donors. We also found that despite greater variability in responses to individual S1-derived epitopes, the magnitude of the response to these peptides was similar to the magnitude of membrane-specific T-cell responses in samples from individual donors. Notably, trends distinguishing mild and moderate SARS-CoV-2 infection could be discerned, although the small number of individuals in each group limits their signficance (mild: *N* = 11; moderate: *N* = 4). In mild cases, three peptides are recognized on average by each individual, whereas eight to nine are recognized per moderate donor (Supplementary Table 3). In addition, the magnitude of responses in moderate cases on average is greater than mild.

Interestingly, the quality of each donor’s cumulative response could be used to distinguish three distinct immunophenotypes within the cohort. These cohorts were defined by whether individuals demonstrated a (1) robust, (2) weak, or (3) no T-cell response. Furthermore, divergent trends in terms of responses could be identified for males and females (Fig. 2b). Although similar T-cell response levels were observed in samples from younger individuals evaluated in this study, regardless of sex, they diverge with age with higher effector T-cell responses in males only (*R* = 0.757, *p* = 0.011, *n* = 7); conversely, older women (>50 years) primarily exhibit decreased T-cell activity. Correlation analysis in the complete cohort of women did not achieve significance (*R* < 0.0001, *p* = 0.993, *n* = 8) due to two outliers (both moderate cases; pneumonia without requiring supplemental oxygen or hospitalization) and possibly because of the small size of the study. However, in the absence of these outliers, the cohort of women with mild cases shows a significant correlation between effector T-cell responses and age (*R* = 0.704, *p* = 0.037, *n* = 6). Collectively, these findings highlight the high prevalence of membrane-targeted T-cell responses in most COVID-19 cases and demonstrates the importance of targeting spike in SARS-CoV-2 vaccination to hasten and focus T-cell responses to this antigen upon infection.

### Pre-existing SARS-CoV-2 immunity in healthy donors is stimulated by predicted epitopes following antigen-specific cell expansion

Although convalescent donors as a group recognized a majority of predicted epitopes ex vivo, as individuals, each recognized only a smaller subset. We hypothesized that SARS-CoV-2-induced T-cell clones underwent variable expansion and contraction over the course of disease, resulting in memory T-cell population frequencies that were both detectable and undetectable ex vivo. To uncover low-frequency epitope-specific T cells, PBMCs were stimulated with SARS-CoV-2 peptides and expanded in short-term culture, and then restimulated with individual or pools of peptides in an IFNγ Fluorospot assay. Although changes to cellular phenotypes over the course of in vitro expansion do not represent the natural immune response to infection, expansion of epitope-specific T cells present ex vivo and their detection by cultured Fluorospot assay may augment the repertoire of immunogenic SARS-CoV-2 T-cell epitopes and suggests greater T-cell memory is generated than thought from ex vivo recall alone.

**Fig. 3:**
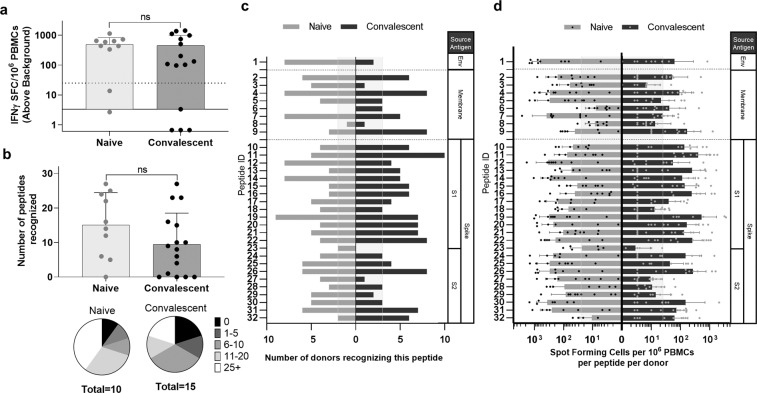
Antigen-specific T-cell expansion increases responses in COVID-19 convalescents and uncovers pre-existing SARS-CoV-2 immunity in healthy donors. **a** PBMCs of convalescent (*n* = 15) and naive donors (*n* = 10) were restimulated with the total peptide pool following an 8-day expansion culture and IFNγ-producing cells were measured by Fluorospot assay. Dotted horizontal line indicates positivity criteria at SFC/10^6^ splenocytes = 25, solid horizontal line indicates threshold of detection for assay (*p* = 0.8216, *t* = 0.2281, d.f. = 23). **b** IFNγ responses to individual peptides were also assessed, identifying the breadth of response in individual donors (*p* = 0.1424, *t* = 1.571, d.f. = 23), **c** the frequency of responses to unique peptides within the cohort (vertical lines denote 20% of each cohort), and **d** the depth of response indicated by the frequency of IFNγ-producing, epitope-specific cells (vertical lines indicate positivity criteria at SFC/10^6^ splenocytes = 25). Error bars depict SD.

Using the same criteria defining an IFNγ response as above, we found that 11/15 (73.3%) convalescent donors had T-cell responses to the pool of 32 peptides (Fig. 3a). Similar to the ex vivo assay, stimulation with individual peptides showed positive responses to envelope and membrane peptides, as well as spike peptides covering the S1 and S2 domains and RBD (Fig. 3d). Only a single S2-derived epitope was not recalled. Samples from convalescent donors recognized between 0 and 27 peptides with an average of 9–10 peptide responses per donor (Fig. 3b). T cells from at least 1 convalescent donor recognized 31/32 (96.9%) peptides, with the majority of peptides (26/32; including 0/1 envelope epitope, 6/8 membrane epitopes, and 20/23 spike epitopes) being confirmed in ≥20% of our cohort. (Fig. 3c).

Changes in IFNγ responses between ex vivo and cultured assay measurements identify broader patterns of peptide recognition associated with source antigen (Supplementary Fig. 1 and Supplementary Table 4). For example, responses identified in convalescent donors to envelope and membrane-derived antigens were rarely replicated following culture in samples from donors that exhibited positive responses ex vivo. In contrast, responses to spike-derived peptides were primarily identified following culture; however, those that did respond ex vivo were much more likely to maintain positive responses following culture as well. These differences may reflect variable memory phenotypes (effector vs. central), proliferative capacities, and/or activation/exhaustion profiles of epitope-specific T-cell populations following natural infection.

Samples from healthy donors demonstrated strong recall responses to SARS-CoV-2 T-cell epitopes following expansion culture, in sharp contrast to the ex vivo response. To the pool of 32 peptides, detectable IFNγ responses were observed in samples from 8/10 (80%) healthy donors (Fig. 3a). To individual peptide restimulations, cross-reactive T-cell responses were found to envelope, membrane, and spike S1, S2, and RBD-derived peptides (Fig. 3d). On average individual control donors responded to 15 peptides, ranging from 0 to 27 recognized peptides per donor sample (Fig. 3b). Following culture, 31/32 (96.9%) peptides stimulated responses in samples from at least one control donor. Of these, only a single peptide was not confirmed in ≥20% of the healthy cohort (Fig. 3c). The 30 peptides correspond to 1/1 envelope peptide, 6/8 membrane peptides, and 23/23 spike peptides. Overall, the magnitude and prevalence of cross-reactive T-cell responses in donors with no SARS-CoV-2 history suggests T-cell memory to CCC infection may contribute to immune response to SARS-CoV-2 infection.

### SARS-CoV-2 peptide vaccination stimulates type 1 immunity

Confirmation that these predicted SARS-CoV-2 peptides are recognized by T cells raised in infection suggested that they may elicit de novo immune responses by vaccination. Peptide vaccination may prime CD4^+^ and CD8^+^ T cells, and generate immune memory that is recalled upon infection, to support protective humoral and cellular mechanisms of immunity. Of the 32 peptides, we selected the highest EpiMatrix scoring peptides (above 20) for vaccination (one peptide was removed due to poor solubility in the pool and was replaced with the 21st ranked peptide). Twenty peptides is in range with what is used in peptide vaccine clinical trials^34^. Peptides were formulated with poly-ICLC (Hiltonol), an adjuvant composed of carboxymethylcellulose, poly-inosinic-poly-cytidylic acid, and poly-l-lysine double-stranded RNA, which stimulates the TLR-3 and MDA-5 innate immune pathways and Th1-skewed CD4^+^ T-cell responses^35^.

HLA-DR3 transgenic mice were primed and boosted with the peptide vaccine (EPV-CoV-19) to assess in vivo vaccine immunogenicity in the context of human major histocompatibility complex (MHC) restriction, which is not feasible using wild-type mice^36^. MHC class II-restricted cellular immunity in the mouse MHC class II knockout/HLA-DR3 knock-in transgenic strain is restricted solely by human MHC class II, not by its murine ortholog, and presents peptides that are also recognized by human T cells. Thus, vaccine immunogenicity in the HLA-DR3 mouse model may support human application of SARS-CoV-2 peptides. Mice were immunized by the intradermal route. A published peptide vaccine clinical trial utilizing poly-ICLC adjuvant and intradermal route of immunization reported minimal injection site reactions and no toxicity associated with the immunizations^37^.

**Fig. 4:**
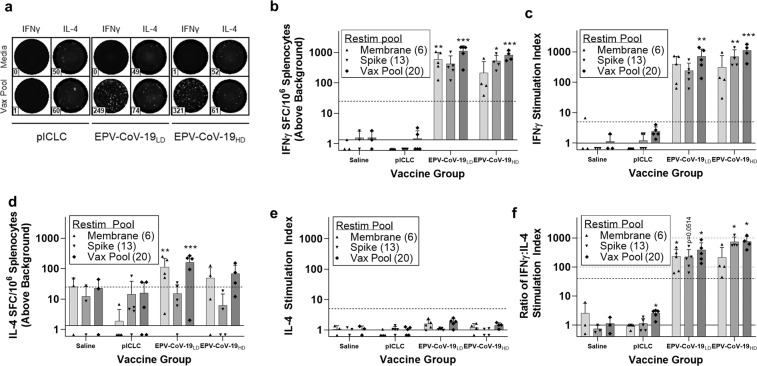
EPV-CoV-19 immunization stimulates strong type 1-skewed T-cell responses in HLA-DR3 transgenic mice. Eight days post boost, murine splenocytes were isolated and assayed for epitope-specific recall responses. Cells were plated in dual IFNγ/IL-4 Fluorospot plates and restimulated with peptide pools for 48 h. **a** Representative images and spot counts are shown for both cytokines (*n* = 3). **b** IFNγ SFC counts were normalized to 1 × 10^6^ cells and adjusted by background subtraction, and **c** IFNγ SI index was determined by calculating the fold change of individual restimulation replicates over background. **d** IL-4 SFC and **e** SI were similarly calculated. Dotted horizontal lines denote positivity criteria of SFC > 25 and SI > 5, respectively. Solid horizontal lines denote threshold of detection for assay. **f** From the reported IFNγ and IL-4 stimulation indexes, we calculated the IFNγ : IL-4 ratio of each restimulation replicate to model the overall skewing of the immune response, identifying a sharply type 1 skewed phenotype in all vaccinated animals. Dotted horizontal lines identify 40, 100, and 1000-fold skewing of type 1/type 2 response. Solid horizontal line identifies hypothetical balanced response (Th1 = Th2). Error bars depict SD. Saline *n* = 3, pICLC *n* = 5, EPV-CoV-19_LD_ *n* = 5, EPV-CoV-19_HD_ *n* = 4, *p < 0.05, **p < 0.01, ***p < 0.001.

The type 1/type 2 T-cell balance stimulated by vaccination was assessed by measuring cytokine production. IFNγ production was measured as a marker of type 1 responses, and interleukin (IL)-4 and IL-5 as markers of type 2 responses. In an IFNγ/IL-4 dual cytokine Fluorospot assay (Fig. 4a), splenocytes from individual mice were stimulated with pools of all vaccine peptides, as well as membrane-derived peptides or spike-derived peptides in the vaccine. All low- and high-dose vaccinated mice mounted an IFNγ response to each pool, as defined by spot forming cells (SFC) (Fig. 4b) and stimulation index (SI) (Fig. 4c) criteria. Although the magnitude of response to each pool was statistically no different between the low- and high-dose groups, the ratio of spike-specific : membrane-specific IFNγ responses was significantly higher in animals that received the high-dose vaccine. Control mice that received either poly-ICLC only or saline did not respond. IL-4 secretion meeting both positivity criteria was not detected in either immunized or mock-immunized mice (Fig. 4d, e). A ratio of the IFNγ and IL-4 responses was used as an indicator of the type 1/type 2 T-cell balance. For both the low-dose and high-dose vaccine groups, the IFNγ/IL-4 ratio skews strongly toward type 1 (Fig. 4f).

**Fig. 5:**
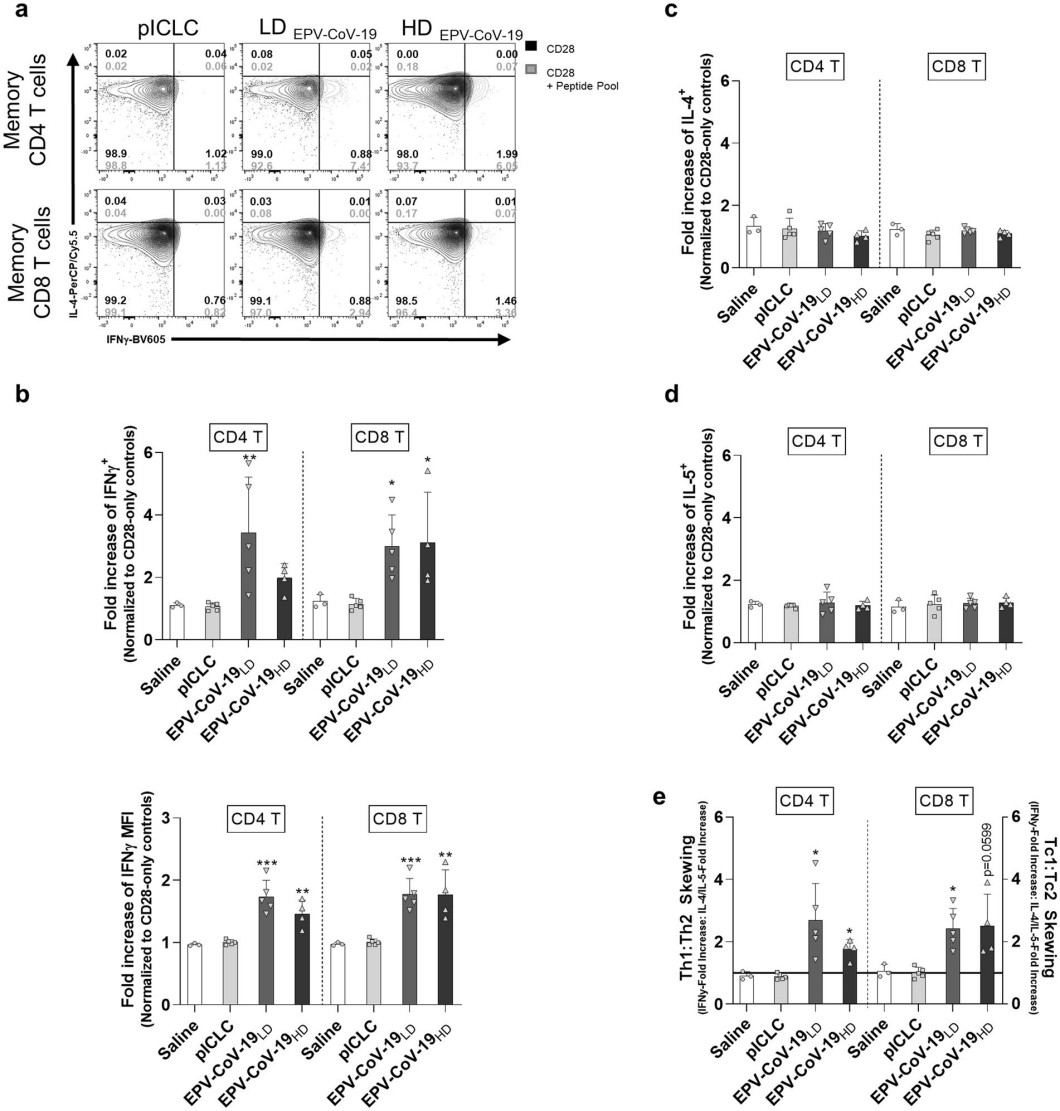
EPV-CoV-19 immunization stimulates type 1-skewed memory CD4^+^ and CD8^+^ T cells in HLA-DR3 transgenic mice. Splenocytes were restimulated with a vaccine-matched peptide pool for 6 h in the presence of brefeldin A and monensin. Following incubation, cells were stained for surface markers, fixed and permeabilized, stained for intracellular markers, and expression of markers was recorded by flow cytometry. Memory CD4^+^ T cells and CD8^+^ T cells were assessed for IFNγ, IL-4, or IL-5-production (both frequency in parent T-cell population and mean fluorescence intensity (MFI) of cytokines). **a** Representative images of type 1 and type 2 skewed, epitope-specific memory T-cell populations are shown. **b** The fold increase of epitope-specific responses (over anti-CD28 stimulated controls) identify vaccine-specific induction of IFNγ, but not **c** IL-4 or **d** IL-5. **e** From ICS generated data, we calculated the fold increase of IFNγ or IL-4- and/or IL-5-producing cells with peptide restimulation, and used the ratio of type 1-to-type 2 responses to model Th-skewing and Tc-skewing in vaccinated animals. Error bars depict SD. Saline *n* = 3, pICLC *n* = 5, EPV-CoV-19_LD_ *n* = 5, EPV-CoV-19_HD_ *n* = 4, *p < 0.05, *p < 0.01, ***p < 0.001.

To assess cytokine production in T-cell subpopulations, we used intracellular cytokine staining and flow cytometry to measure frequency, mean fluorescence intensity, and differentiation status of splenic CD4^+^ and CD8^+^ T cells responding to EPV-CoV-19 peptides (Fig. 5a and Supplementary Fig. 2). EPV-CoV-19 vaccination at both low and high doses stimulated statistically significant increases in numbers of IFNγ-producing memory T cells that were recalled specifically by vaccine peptides, as well as increased average amounts of IFNγ produced per cell compared to control mice (Fig. 5b and Supplementary Fig. 3). In contrast, neither IL-4 nor IL-5 production from memory CD4^+^ and CD8^+^ T cells above control levels could be restimulated by vaccine peptides in vitro (Fig. 5c, d). Although we do note a minimal increase in the frequency of IL-5-producing CD4^+^ T cells from vaccinated animals (Supplementary Fig. 3), there is no concurrent increase in IL-5 production per cell nor is the magnitude of this response comparable to the induction of Th1 subsets, thus minimizing the functional significance of this observation.

Overall, both the low-dose and high-dose vaccine groups exhibit IFNγ/(IL-4 + IL-5) ratios that sharply skew toward Th1/Tc1 (Fig. 5e). Taken altogether, these results show EPV-CoV-19 stimulates a strong T-cell response that can be recalled to elicit a potent type 1-skewed response in a dose-independent manner, while also critically avoiding induction of significant type 2 responses associated with enhanced respiratory disease.

## Discussion

SARS-CoV-2 infection causes a broad range of disease, known as COVID-19, from mild or no symptoms to serious complications that may be rapidly fatal, often in adults over 65 years old and individuals with underlying medical conditions, including cardiovascular disease, type 2 diabetes, and obesity^38,39^. Development of effective, safe, and well-accepted vaccines is crucial in controlling the current pandemic.

Our study identifies SARS-CoV-2 T-cell epitopes that are recognized in natural infection and stimulate pre-existing immunity to SARS-CoV-2. The study was limited to mild and moderate COVID-19 cases, to identify T-cell epitopes recognized in the protective immune response that could form the basis for T-cell-directed vaccines. Although neutralizing antibody-targeted SARS-CoV-2 vaccines based on the spike antigen are approved for emergency use and will be the near-term approach for global immunization, they may not be sufficiently effective at controlling viral infection and replication in the upper respiratory tract, and therefore may not be as effective at controlling viral transmission as they are at controlling disease. Furthermore, the durability of protection afforded by these vaccines and their ability to prevent asymptomatic infection are unknown. Moreover, the virus may evade antibody-mediated immunity. The receptor-binding motif is the least conserved part of the spike antigen and there is already evidence of virus escape from neutralizing monoclonal antibodies without vaccine pressure^40^.

These concerns open up space for T-cell-focused vaccine development, even though it is not expected that a T-cell vaccine would replace antibody-directed vaccines. A T-cell targeting vaccine composed of conserved epitopes may provide rapid, effective, and long-term immunity at sites of infection through the generation of tissue resident memory CD8^+^ T cells, as well as memory CD4^+^ T cells that support antibody responses. Influenza vaccination during the 2009 H1N1 pandemic demonstrated that memory CD4^+^ T cells are able to support naive B-cell responses to a novel hemagglutinin^41–43^. A T-cell-directed SARS-CoV-2 vaccine could generate robust CD4^+^ T-cell memory that would provide early control of acute infection with a novel SARS-CoV-2 or closely related virus in the absence of pre-existing cross-protective antibodies.

In addition to vaccine applications, finely mapped T-cell epitopes can be used to address outstanding questions about T-cell immunity in COVID-19 such as the relationship between memory T-cell development and protective antibody durability, the role of T cells in prolonged COVID-19, the type of T-cell response that predicts vaccine protection, and the possible T-cell involvement in early protective efficacy after one dose of the Pfizer/BioNTech, Moderna, and AstraZeneca/Oxford vaccines before neutralizing antibody is observed^44–46^, and the role of pre-existing SARS-CoV-2 T-cell immunity in infection and vaccination. With respect to the latter idea, we found healthy donors with no history of SARS-CoV-2 exposure displayed IFNγ responses to SARS-CoV-2 epitopes, as has been observed in other studies^21,47,48^. Although responses were almost exclusively undetected ex vivo, strong responses to 31/32 epitopes were found after culture. These epitopes may be used in longitudinal studies that track T-cell responses before and after SARS-CoV-2 infection and vaccination, to assess whether cross-reactive T-cell memory confers an advantage for rapid response upon exposure or vaccination that might offer a measure of protection otherwise unavailable to a naive host. It has been hypothesized that pre-immunity may result from prior exposure to related coronaviruses that cause the common cold, and epidemiological studies have shown that COVID-19 risk and severity are reduced in persons with recent CCC infection^49,50^. These findings further motivate investigation into the role cross-reactive CD4^+^ and CD8^+^ T cells may play in protective immunity.

Sequence relatedness of SARS-CoV-2 and CCC spike and membrane antigens studied here suggest potential for cross-reactivity. Indeed, Mateus et al.^51^ found that SARS-CoV-2 peptides with >67% homology with the corresponding CCC peptides were associated with cross-reactivity. We found the TCR face of SARS-CoV-2 HLA class II epitopes demonstrated no homology with CCCs when requiring all positions available to the TCR to be identical. However, when this constraint was loosened, several epitopes exhibited homology that is compatible with the immune responses we observed in vitro. Notably, Mateus et al.^51^ did not identify cross-reactivity in the membrane antigen, while here we find six SARS-CoV-2 membrane epitopes stimulate responses in healthy donors. This discrepancy might be explained by differences in the methods used to expand antigen-specific T cells or in the immunoinformatic methods used to select epitopes for testing in the two studies.

A number of groups have identified immunoreactive SARS-CoV-2 T-cell epitopes using either epitope predictors or arrays of overlapping peptides^51–53^. Our studies confirmed 18 epitopes and discovered 14 previously unpublished epitopes (Table 4). Confirmed epitopes include 1 membrane and 1 spike epitope found by Nelde et al.^52^ who predicted CD4^+^ and CD8^+^ T-cell epitopes and measured T-cell responses to individual peptides in cultured ELISpot assays using PBMCs from SARS-CoV-2 convalescent and naive donors, 3 membranes and 5 spike epitopes by Peng et al.^53^ who performed ex vivo ELISpot stimulations of cells from SARS-CoV-2 convalescents using overlapping peptides, and 19 spike epitopes by Mateus et al.^51^ who measured responses to predicted epitopes by cultured ELISpot assay.

**Table 4:** Published SARS-CoV-2 surface antigen epitopes^a^.

Input sequence	Cluster address	Cluster sequence	Nelde et al^52^.	Peng et al^53^.	Mateus et al^51^.
MEMBRANE	146–160	RGHLRIAGHHLGRCD		GAVIL**RGHLRIAGHHLGR LRIAGHHLGRCD**IKDLPK	
MEMBRANE	165–179	PKEITVATSRTLSYY		**PKEITVATSRTLSYY**KL	
MEMBRANE	175–190	TLSYYKLGASQRVAGD	**LSYYKLGASQRVAGD**	TSR**TLSYYKLGASQRVA**	
MEMBRANE	201–217	IGNYKLNTDHSSSSDNI		**IGNYKLNTDHSSSSDNI**A	
SPIKE	140–156	FLGVYYHKNNKSWMESE			**LGVYYHKNNKSWMES**
SPIKE	198–212	DGYFKIYSKHTPINL			NI**DGYFKIYSKHTPI**
SPIKE	239–255	QTLLALHRSYLTPGDSS	ITRF**QTLLALHRSYL**		TRF**QTLLALHRSYLT LLALHRSYLTPGDSS**
SPIKE	315–329	TSNFRVQPTESIVRF			GIYQ**TSNFRVQPTES SNFRVQPTESIVRF**P **QPTESIVRF**PNITNL
SPIKE	338–352	FGEVFNATRFASVYA			CP**FGEVFNATRFASV VFNATRFASVYA**WNR
SPIKE	347–364	FASVYAWNRKRISNCVAD		**YAWNRKRISNCVAD**Y	R**FASVYAWNRKRISN**
SPIKE	446–468	GGNYNYLYRLFRKSNLKPFERDI		**GGNYNYLYRLFRKSN**	**GGNYNYLYRLFRKSN**
SPIKE	483–500	VEGFNCYFPLQSYGFQPT			**FNCYFPLQSYGFQPT**
SPIKE	797–816	FGGFNFSQILPDPSKPSKRS		**NFSQILPDPSKPSKR**	**NFSQILPDPSKPSKR**
SPIKE	866–879	TDEMIAQYTSALLA		**TDEMIAQYTSALLA**G	**TDEMIAQYTSALLA**G **AQYTSALLA**GTITSG
SPIKE	895–911	QIPFAMQMAYRFNGIGV			**IPFAMQMAYRFNGIG QMAYRFNGIGV**TQNV
SPIKE	998–1012	TGRLQSLQTYVTQQL			LI**TGRLQSLQTYVTQ**
SPIKE	1152–1166	LDKYFKNHTSPDVDL			E**LDKYFKNHTSPDVD**
SPIKE	1169–1183	ISGINASVVNIQKEI		**GINASVVNIQKEI**DR	**GINASVVNIQKEI**DR

^a^Amino acid regions from published peptides in three early publications that overlap with T-cell epitopes in this study are bold and underlined.

Although there is significant overlap between our spike, membrane, and envelope epitope set with others, we found several epitopes that have hitherto gone undetected. One reason that our results may differ from these studies could be that we focused selection on epitopes that are highly promiscuous (bind to multiple HLA DR alleles) and we avoided inclusion of T-cell epitopes that are conserved with the human genome. Of the 52 epitope clusters identified in SARS-CoV-2 spike, membrane, and envelope, 17 (32.7%) have elevated regulatory T-cell induction potential. This suggests that effector and regulatory CD4^+^ T-cell subsets may be activated by these antigens in the course of SARS-CoV-2 infection, resulting in induction of regulatory T cells that may reduce in vitro measurements of effector T-cell recall. Exclusion of such sequences from the set of effector T-cell epitopes tested here may have contributed to a higher overall positivity rate. This has implications for vaccine design. Engineering out human-like CD4^+^ T-cell epitopes from spike may improve the T-cell response supporting the quality of B-cell responses and durability of neutralizing antibody levels.

In summary, we assessed the T-cell immunogenicity potential of SARS-CoV-2 spike, membrane, and envelope antigens using a computational approach to quickly identify virus sequences that stimulated effector T cells, while setting aside those that might stimulate regulatory T cells in infection and vaccination. T-cell epitope prediction using computational tools has progressed over close to three decades and has become an integral part of vaccine development programs for antigen discovery, epitope mapping, and vaccine design. Advances in the field have made it possible to rapidly identify and design vaccine candidates in situations requiring a speedy response, including cancer diagnosis and novel infectious disease outbreaks, be they natural, accidental, or deliberate. We intend to move the epitopes identified here to clinical trials in collaboration with our partners.

## Methods

### SARS-CoV-2 sequences

SARS-CoV-2 (taxid: 2697049), SARS-CoV-1 (taxid: 694009), MERS-CoV (taxid: 1335626), and human CoV (taxids: 11137, 443239, 277944, and 31631) antigen sequences isolated from human hosts were obtained from GenBank at the National Center for Biotechnology Information. SARS-CoV-2 epitopes were compared across sequences obtained from isolates with fully sequenced genomes isolated from January 2020 to January 2021. SARS-CoV-2 Wuhan-Hu-1 (GenBank id: MN908947) was selected as the reference strain.

### T-cell epitope mapping

Each antigen sequence was parsed into all possible linear 9-mer sequences. Each 9-mer was scored for likelihood of binding to panels of HLA class I and class II alleles using EpiMatrix version 1.3, a matrix-based algorithm for mapping T-cell epitopes. Class II epitopes were identified for nine supertype alleles as follows: DRB1*0101, DRB1*0301, DRB1*0401, DRB1*0701, DRB1*0801, DRB1*0901, DRB1*1101, DRB1*1301, and DRB1*1501. Class I epitope 9-mers (and 10-mers) were identified for potential binding to six supertype alleles as follows: A*0101, A*0201, A*0301, A*2402, B*0702, and B*4401. Each allele set covers >95% of the human population^22,23^. Promiscuous HLA class II epitopes representing regions of a protein with a high density of T-cell immunogenicity potential were identified using the ClustiMer algorithm. Clusters scoring an overall EpiMatrix score ≥ 10 after subtracting the average expected sum of scores for a random sequence of equal length have significant immunogenicity potential^20^.

### T-cell epitope conservation analysis

The JanusMatrix algorithm^24^ was used to identify SARS-CoV-2 epitopes that share TCR-face conservation (positions 2, 3, 5, 7, and 8) with epitopes restricted by the same alleles, but found in the human proteome, and other (α- and β-) coronavirus epitopes. Epitopes with identical TCR-facing residues, which are also predicted to bind to the same MHC allele, are more likely to induce cross-reactive T cells. A JanusMatrix homology score threshold of two (cross-conserved HLA allele-specific epitopes averaged over the length of the sequence) for cross-conservation with human (self) proteins was applied to identify epitopes with elevated potential to be tolerated or actively regulatory. To investigate the conservation of SARS-CoV-2 epitopes with related highly pathogenic coronaviruses (SARS-CoV and MERS-CoV) and low-pathogenicity CCCs (OC43, HKU1, NL62, and 229E), JanusMatrix was applied requiring identical TCR-facing residues at five positions and at positions 5 and 8 only. JanusMatrix homology scores calculated for cross-conservation with coronaviruses were scaled proportionally to the number of sequences in each dataset. A separate conservation analysis of iVAX-predicted SARS-CoV-2 T-cell epitopes with published coronavirus epitopes deposited in the Immune Epitope Database was performed using BLAST and a cutoff of 80% similarity.

### Peptide synthesis

Synthetic peptides were manufactured using 9-fluoronylmethoxycarbonyl chemistry by 21st Century Biochemicals (Marlboro, MA). Peptide purity was >90% as ascertained by analytical reversed-phase high-performance liquid chromatography. Peptide mass was confirmed by tandem mass spectrometry.

### SARS-CoV-2 convalescent donors

Convalescent patients were recruited by Sanguine Biosciences, a clinical services group that identified, consented, and enrolled participants. Inclusion criteria included subjects (i) willing and able to provide written informed consent; (ii) aged 18–80 years of age, both male or female; and (iii) PCR-confirmed COVID-19 diagnosis (recovered) with date of diagnosis a minimum of 30 days from blood collection. Exclusion criteria included subjects who (i) were pregnant or nursing; (ii) had a known history of HIV, hepatitis, or other infectious diseases; (iii) had autoimmune diseases; (iv) were members of vulnerable patient population (prisoners, mentally impaired); (v) had medical conditions impacting their ability to donate blood (i.e., anemia, acute illness); (vi) had received immunosuppressive therapy or steroids within the last 6 months; (vii) had received an investigational product in the last 30 days; (viii) had experienced excess blood loss including blood donation defined as 250 mL in the last month or 500 mL in the last two months; or (ix) had a positive COVID-19 PCR test, but were asymptomatic. Samples were collected in accordance with NIH regulations and with the approval of an independent external institutional review board (Ethical & Independent Review Services, Independence, MO).

### Healthy unexposed donors

Deidentified samples were obtained from leukocyte reduction filters from the Rhode Island Blood Center for unrelated studies prior to the SARS-CoV-2 outbreak in December 2019 (date of samples: February 2016–November 2019). Samples were obtained in accordance with NIH regulations and with the approval of an independent external institutional review board (Ethical & Independent Review Services, Independence, MO).

### PBMC culture

PBMCs from COVID-19 convalescents were put into culture directly following Ficoll separation. Cells from normal healthy donors were thawed and rested overnight before placing into culture. Samples were allocated for ex vivo assay and antigen-specific cell expansion followed by cultured assay. Antigen-stimulated cells were cultured over 8 days at 37 °C under a 5% CO_2_ atmosphere. In a 48-well plate, 5 × 10^6^ cells in 150 μL RPMI medium supplemented with human AB serum were stimulated with pools of peptides at 10 μg/mL on Day 1. Three days later, IL-2 was added to 10 ng/mL and the culture volume raised to 300 μL. On Day 7, cells were supplemented with 10 ng/mL IL-2 by half media replacement. Two days later, PBMCs were collected and washed in preparation for measurement of immune recall responses.

### Ex vivo and cultured human Fluorospot assay

IFNγ Fluorospot assays were performed using ex vivo and cultured PBMCs using assay kits purchased from Mabtech, and were executed according to the manufacturer’s specifications. For ex vivo assays, unless otherwise noted, peptides were added individually at 20 μg/mL or pooled at 10 μg/mL per peptide in triplicate wells containing 250,000 PBMCs in RPMI medium supplemented with 10% human AB serum. For cultured assays, peptides were added individually at 10 μg/mL or pooled at a total peptide concentration of 10 μg/mL (32 peptides, 0.313 μg/mL) and added to triplicate wells containing 100,000 PBMCs. Triplicate wells were plated with Concanavalin A (ConA; 5 μg/mL) as a positive control and six wells containing no antigen stimulus (0.2–0.4% dimethyl sulfoxide (DMSO)) were used for background determination. Cells were incubated for 40–48 h at 37 °C under a 5% CO_2_ atmosphere. Plates were developed according to the manufacturer’s directions using fluorescein isothiocyanate (FITC)-labeled anti-IFN-γ detection antibody.

Raw spot counts were recorded by ZellNet Consulting, Inc., using a FluoroSpot reader system (iSpot Spectrum, AID, Strassberg, Germany) with software version 7.0, build 14790, where fluorescent spots were counted utilizing FITC and Cy3 filters. Camera exposure and gain settings were adapted for each filter to obtain high-quality spot images preventing over- or underexposure. Fluorophore-specific spot parameters were defined using spot size, spot intensity, and spot gradient (fading of staining intensity from center to periphery of spot), and a spot separation algorithm was applied for optimal spot detection.

Results were calculated as the average number of spots in the peptide wells, adjusted to spots per one million cells. Responses meeting the following criteria were considered to be positive when the number of spots was (i) at least 5 times background, (ii) >25 spot-forming cells per well above background (1 response per 40,000 PBMCs), and (iii) statistically different (*p* < 0.05) from the media-only control by the Student’s *t*-test.

### HLA typing

Donor HLA class II haplotypes were determined using the One Lambda Micro SSPTM High Resolution HLA class II kit at the Hartford Hospital Transplant Immunology Laboratory.

### Mice

HLA-DR3 transgenic mice were obtained from Dr. Chella David (Mayo Clinic) under commercial license. The mice express the *HLA-DRA* and DRB1*0301 genes on a B.10-Ab^0^ mouse class II-negative background. Animal research protocols for mouse studies were reviewed and approved by the Absorption Systems, Inc., Institutional Animal Care and Use Committee.

### Peptide vaccine preparation

Per dose, a pool of 20 peptides at 1.25 or 5.0 μg/peptide was admixed with 50 μg poly-ICLC (Hiltonol^TM^; Oncovir) in 50 μL.

### Vaccinations

Vaccine- and sham-treated HLA-DR3 mice (*N* = 5/group) were female and 6–8 weeks old at the start of immunizations. Mice were primed and boosted 2 weeks later by intradermal immunization with peptide/poly-ICLC vaccine. Control groups received sterile water (*N* = 3) or poly-ICLC alone (*N* = 5). Mice were killed 9 days after the boost immunization. Blood at baseline and termination, and the spleens were collected for immune monitoring. One mouse in the group that received 5 μg/peptide was excluded following splenocyte isolation due to insufficient recovery and poor viability for reasons thought to be unrelated to vaccination.

### Ex vivo FluoroSpot assay in mouse splenocytes

The frequency of vaccine-specific splenocytes was determined by dual cytokine IFNγ/IL-4 FluoroSpot assay using the Mabtech mouse IFNγ/IL-4 FluoroSpot Kit with pre-coated plates according to the manufacturer’s protocol. Washed splenocytes in RPMI 1640 (Gibco) supplemented with 10% fetal calf serum (Atlanta Biologicals) were added at 250,000 cells per well. Antigen stimulations included pools of all 20 vaccine peptides, as well as spike-derived vaccine peptides and membrane-derived vaccine peptides. Peptide pools were added at 0.5 μg/mL per peptide. Triplicate wells were stimulated with 2 μg/mL ConA (Sigma Aldrich) as a positive control and six replicate wells with medium containing 0.2% DMSO were used for background determination. Raw spot counts were recorded by ZellNet Consulting, Inc., and results were calculated as described above for human FluoroSpot assays.

### Flow cytometry in mouse splenocytes

Splenocytes were plated at 300,000 cells per well and stimulated in triplicate over 6 h with a pool of all 20 vaccine peptides at 0.5 μg/mL per peptide and 4 μg/mL co-stimulatory anti-CD28 antibody. Triplicate wells were stimulated with phorbol myristate acetate (50 ng/mL) + ionomycin (1 μg/mL) as a positive control. For background determinations, triplicate wells were treated with medium containing 0.2% DMSO only and medium containing 0.2% DMSO and 4 μg/mL co-stimulatory anti-CD28 antibody. Brefeldin A (5 ng/μL) and 2 μM monensin were added with stimulations to enable detection of intracellular cytokines. Following stimulation, cells were incubated with Fixable Viability Stain 450 to discriminate dead from live cells and then stained with the following surface marker antibody panel: CD3e-AF700 (clone 500A2), CD4-APC/Fire750 (clone GK1.5), CD8a-FITC (clone 53-6.7), CD62L-APC (clone MEL-14) (BioLegend), and CD44-eFLuor506 (clone IM7) (Thermo). To detect intracellular cytokine expression, cells were fixed and permeabilized, and immunostained using IFNγ-BV605 (clone XMG1.2), IL-4- PerCP/Cy5.5 (clone 11B11), and IL-5-PE (clone TRFK5) (BioLegend) antibodies. Flow cytometry measurements were made on an Invitrogen Attune cytometer and collected data analyzed using FlowJo software (Version 10.6.2). Cells were gated on lymphocyte/singlet/live events. Recalled Th1 and Th2 cells were defined, respectively, as IFNγ-producing CD3^+^CD4^+^ CD44^+^ T cells and IL-4- and/or IL-5-producing CD3^+^CD4^+^ CD44^+^ T cells. Tc1 and Tc2 cells were defined, respectively, as IFNγ-producing CD3^+^CD8^+^ CD44^+^ T cells and IL-4- and/or IL-5-producing CD3^+^CD8^+^ CD44^+^ T cells.

### Statistical analysis

For human PBMC studies, statistical significance between naive and convalescent donor responses was determined using unpaired *t*-tests (naive *n* = 10, convalescents *n* = 15). To determine a relationship between age and the degree of the SARS-CoV-2-specific IFNy response, Pearson’s correlation was applied (male cohort *n* = 7, female cohort *n* = 8, mild subset of female cohort *n* = 6). For both sets of analyses, *p*-values were determined using two-tailed tests. For vaccine immunogenicity assessment in mice, one-way analysis of variance (ANOVA) was used to evaluate the fold increases in vaccine-induced/peptide-specific response (average response to co-stimulation + matched peptide over response to co-stimulation alone in splenocytes derived from individual animals). For immunogenicity studies comparing multiple restimulation conditions in each of the vaccine groups, two-way ANOVA was applied. For all ANOVA analyses, data were unmatched with multiple comparisons (comparing mean of each vaccine group’s fold increase or pool-specific response to pICLC controls) and corrected using Dunnett’s test (saline *n* = 3, pICLC *n* = 5, EPV-CoV-19_LD_ *n* = 5, and EPV-CoV-19_HD_ *n* = 4). To determine significance for type-1 vs. type-2 skewing of the immune response, significance was determined using a one-sample *t*-test compared to a theoretical mean of 1. For all analyses, significance was attributed to calculated *p*-values < 0.05.

### Reporting summary

Further information on research design is available in the Nature Research Reporting Summary linked to this article.

## Supplementary information

Supplementary Information

Reporting Summary

## Data Availability

Data generated or analyzed during this study that are critical to the reported findings are included in this published article and its Supplementary Information files. Additional supporting data are available from the corresponding author on reasonable request.
