# Supplementary material for: Highly conserved, non-human-like, and cross-reactive SARS-CoV-2 T cell epitopes for COVID-19 vaccine design and validation
Source: NPJ Vaccines. 2021 May 13;6:71. doi: 10.1038/s41541-021-00331-6 (PMC8119491; doi:10.1038/s41541-021-00331-6)
Supplement: Supplementary file 1 — Supplementary Information [file 41541_2021_331_MOESM1_ESM.pdf]

SUPPLEMENTARY INFORMATION

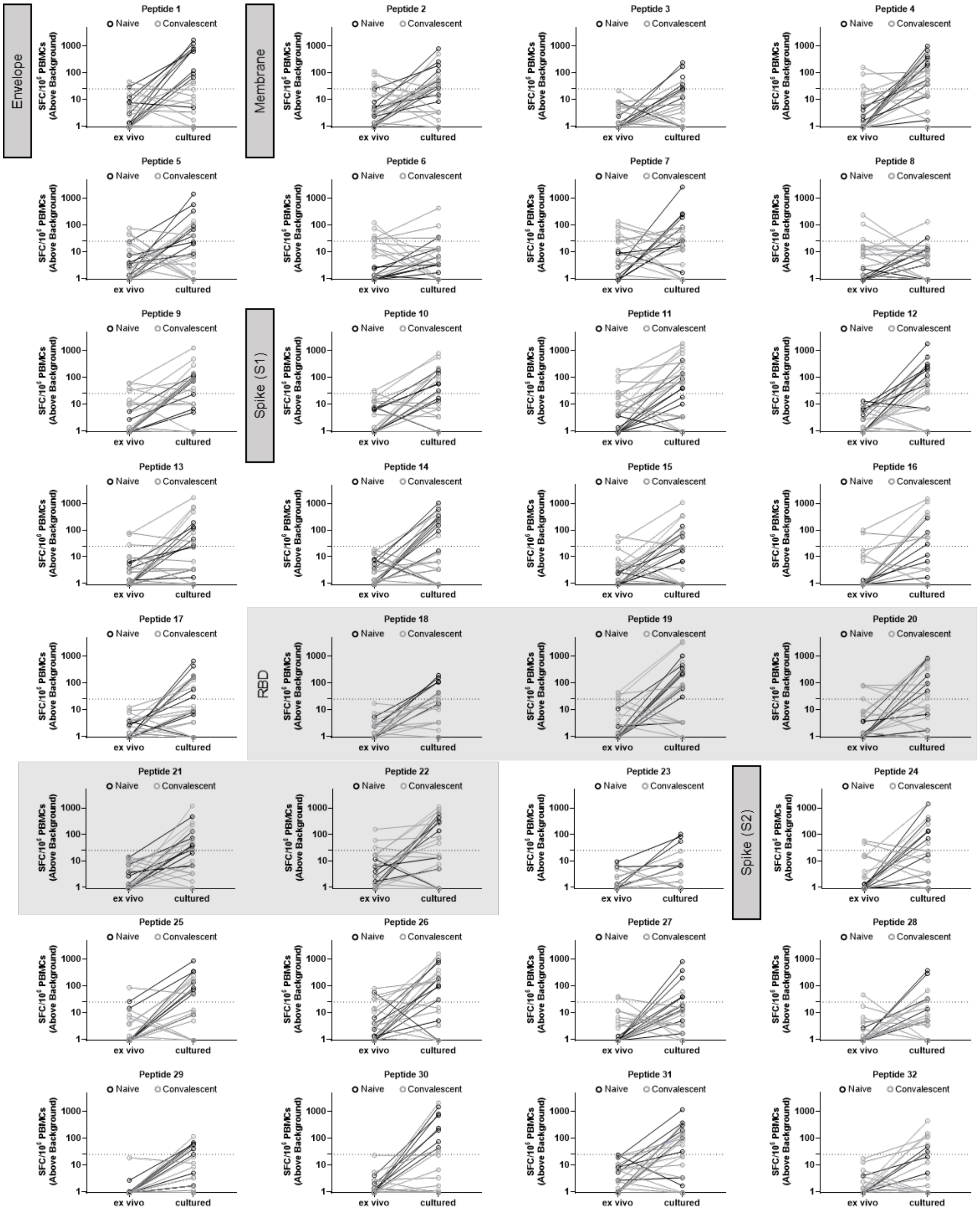

Supplementary Figure 1. Recognition of individual peptides ex vivo and following culture identify broader patterns of antigen-specific responses. Depth of individual donor responses

to unique peptides before and after epitope-specific T cell expansion suggest antigen-specific patterns of immune responses developed during natural infection. These include those that appear ex vivo but cannot be reconfirmed following culture, positive responses that can only be identified following epitope-specific T cell expansion, and those that can be identified ex vivo and maintained following 8-day culture/restimulation. Furthermore, frequency of clones with limited expansion potential (ex vivo response lost after culture) and those requiring expansion for detection (frequency of culture-only) suggest variable T cell phenotypes of naturally expanded, antigen-specific cells.

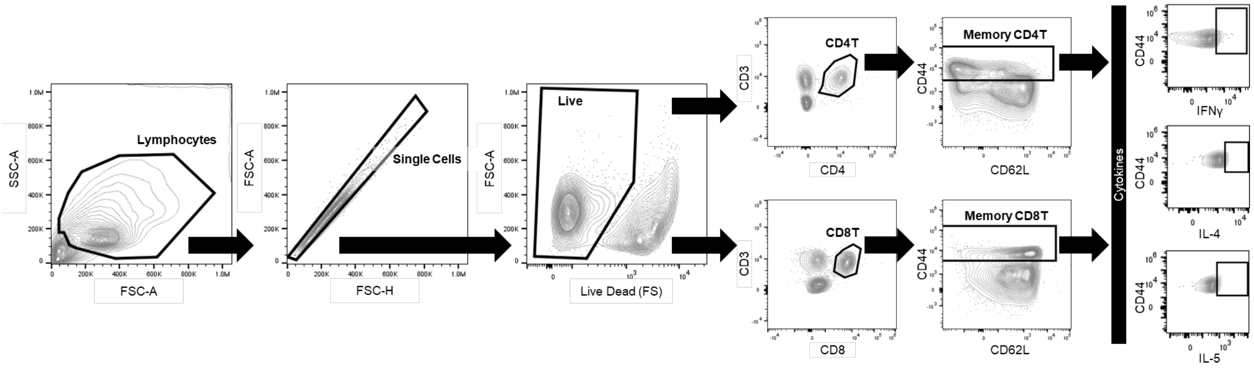

Supplementary Figure 2. Gating scheme for analysis of EPV-CoV-19 immunized mice. Gating of flow cytometry data identifies IFNγ, IL-4 and/or IL-5 production in $CD3^+CD4^+CD44^+$ and $CD3^+CD8^+CD44^+$ T cells.

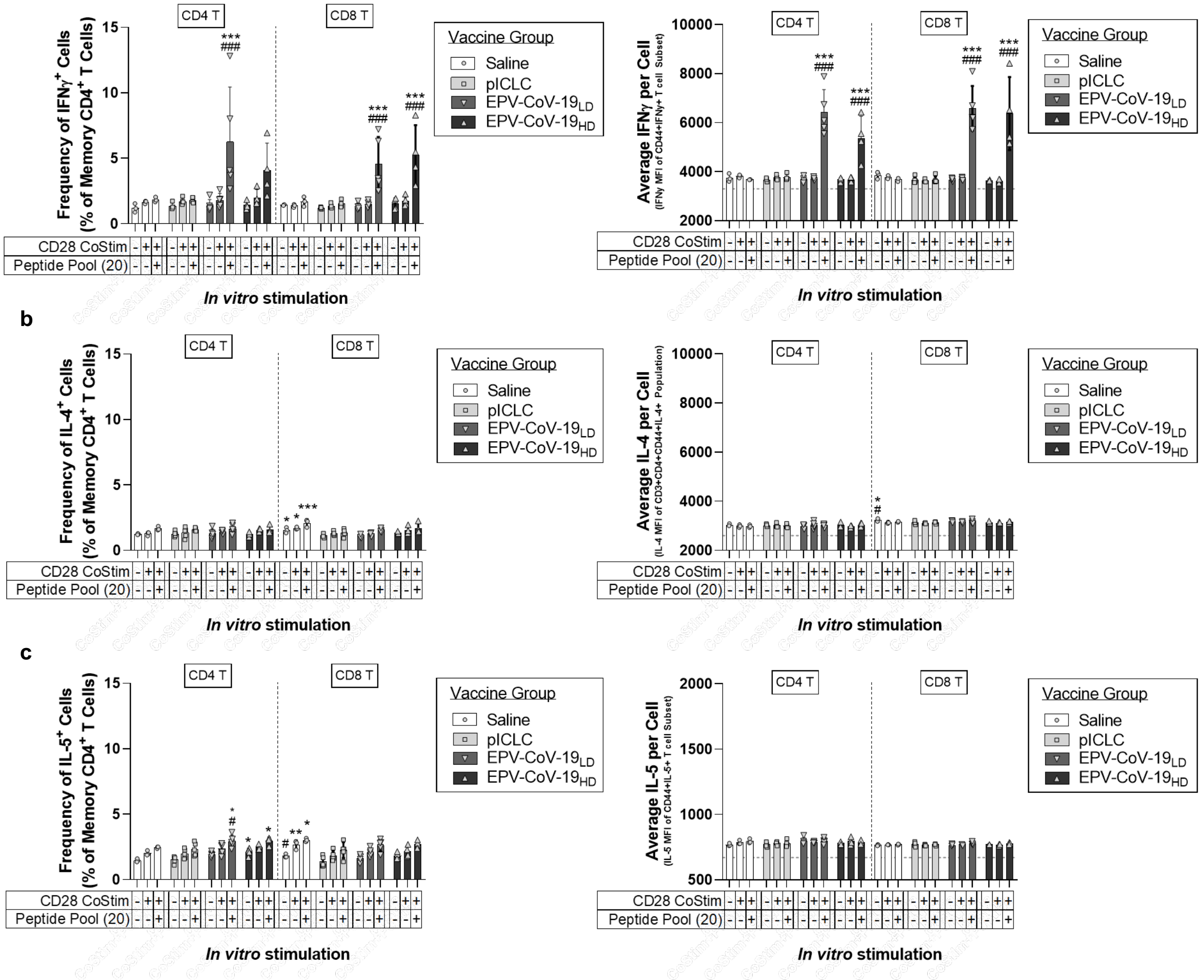

Supplementary Figure 3. Monitoring T cell phenotypes of EPV-CoV-19 immunized mice identifies specific induction of type 1 response. **(a)** ICS confirms the peptide-specific recall of vaccine-induced Th1 and Tc1 memory responses as the frequency of IFNγ^{+} cells, as well as the IFNγ produced per cell, is increased in both T cell subsets. The frequency of **(b)** IL-4 or **(c)** IL-5 producing cells (as well as the MFI of type 2 cytokines in these cells) were also monitored, confirming significant induction of Th1/Tc1 skewed memory in vaccinated animals.

Supplementary Table 1. Predicted class II HLA immunogenicity potential of spike epitope clusters containing mutations found in SARS-CoV-2 variants of concern

Mutation	Reported location	Cluster Address	Cluster Sequence[1]		EpiMatrix Cluster Score		JanusMatrix Human Homology Score		Effect on immunogenicity potential	Effect on human cross-conservation
			Reference	Variant	Reference	Variant	Reference	Variant		
**Lineage B.1.1.7**										
HV69-70 del	UK	52 - 69	QDLFLPFFSNVTWFHAIH	QDLFLPFFSNVTWFHAI	16.27	12.57	0.22	0.22	Decrease	No
HV69-70 del	UK	61 - 79	NVTWFHAIHVSGTNGTKRF	NVTWFHAI--SGTNGTKRF	21.41	14.71	0.54	0.08	Decrease	Decrease
Y145 del	UK	137 - 156	NDPFLGVYYHKNNKSWMESE	NDPFLGVY-HKNNKSWMESE	23.78	17.76	0.00	0.00	Decrease	No
N501Y	UK	492 - 508	LQSYGFQPTNGVGYQPY	LQSYGFQPT**Y**GVGYQPY	10.08	2.67	0.14	0.00	Decrease[2]	Decrease
T716I	UK	715 - 729	PTNFTISVTTEILPV	P**I**NFTISVTTEILPV	12.63	12.63	0.00	0.27	No	Increase
**Lineage B.1.351**										
D215G	SA	207 - 223	HTPINLVRDLPQGFSAL	HTPINLVR**G**LPQGFSAL	14.08	14.49	4.25	4.63	Increase	Increase
LLA241-243 del	SA	232 - 255	GINITRFQTLLALHRSYLTPGDSS	GINITRFQT---LHRSYLTPGDSS	55.01	27.62	1.03	0.78	Decrease[2]	Decrease
E484K	SA	483 - 500	VEGFNCYFPLQSYGFQPT	V**K**GFNCYFPLQSYGFQPT	17.96	17.96	0.00	0.00	No	No
N501Y	SA	492 - 508	LQSYGFQPTNGVGYQPY	LQSYGFQPT**Y**GVGYQPY	10.08	2.67	0.14	0.00	Decrease[2]	Decrease
**Lineage B.1.427**										
S13I	US (CA)	1 - 26	MFVFLVLLPLVSSQCVNLTTRTQLPP	MFVFLVLLPLVS**I**QCVNLTTRTQLPP	71.61	67.22	3.19	3.13	Decrease	Decrease
L452R	US (CA)	442 - 468	DSKVGGNYNYLYRLFRKSNLKPFERDI	DSKVGGNYNY**R**YRLFRKSNLKPFERDI	58.42	43.72	1.03	1.23	Decrease[2]	Increase
L938F	US (CA)	934 - 949	IQDSLSSTASALGKLQ	IQDS**F**SSTASALGKLQ	11.21	16.11	3.11	2.70	Increase	Decrease
**Lineage P.1**										
P26S	Brazil	25 - 39	PPAYTNSFTRGVYYP	P**S**AYTNSFTRGVYYP	12.80	12.80	0.17	0.17	No	No
D138Y	Brazil	137 - 156	NDPFLGVYYHKNNKSWMESE	N**Y**PFLGVYYHKNNKSWMESE	23.78	23.78	0.00	0.00	No	No
E484K	Brazil	483 - 500	VEGFNCYFPLQSYGFQPT	V**K**GFNCYFPLQSYGFQPT	17.96	17.96	0.00	0.00	No	No
N501Y	Brazil	492 - 508	LQSYGFQPTNGVGYQPY	LQSYGFQPT**Y**GVGYQPY	10.08	2.67	0.14	0.00	Decrease[2]	Decrease
T1027I	Brazil	1010 - 1029	QQLIRAAEIRASANLAATKM	QQLIRAAEIRASANLAA**I**KM	25.75	25.75	2.73	2.73	No	No
V1176F	Brazil	1166 - 1183	LGDISGINASVVNIQKEI	LGDISGINAS**F**VNIQKEI	15.12	17.41	1.00	0.50	Increase	Decrease

[1]Amino acid mutations are shown in red. Deletions are shown as dashes.

[2]Significant decrease in immunogenicity potential predicted by EpiMatrix. Decrease in EpiMatrix Cluster Score of more than 10 points between reference and variant or decrease below the cluster threshold of 10 is considered significant.

Supplementary Table 2. Convalescent donor characteristics

**BMI**	
**Range**	21.1-43.4
**Obese**	13.33% (2/15)
**Overweight**	46.67% (7/15)
**Normal**	40% (6/15)
**Underweight**	0
**Symptoms**	
**Fever**	73.33% (11/15)
**Fever Duration (days)**	1.5-14
**Cough**	80% (12/15)
**Shortness of breath**	46.67% (7/15)
**Muscle pain**	80% (12/15)
**Headache**	93.33% (14/15)
**Sore throat**	53.33% (8/15)
**Loss of taste or smell**	73.33% (11/15)
**Nausea**	26.67% (4/15)
**Weight Loss**	6.67% (1/15)
**Loss of appetite**	20% (3/15)
**Diarrhea**	40% (6/15)
**Urinary Incontinence**	6.67% (1/15)
**Fatigue**	20% (3/15)
**Malaise**	6.67% (1/15)
**Sweats**	20% (3/15)
**Chills**	20% (3/15)
**Dizziness**	13.33% (2/15)
**Pneumonia**	13.33% (2/15)
**Anemia**	6.67% (1/15)
**Sensitivity to light**	6.67% (1/15)
**Peeling Skin**	6.67% (1/15)
**Chest Pain**	6.67% (1/15)
**Back Pain**	6.67% (1/15)
**Numbing of foot**	6.67% (1/15)
**Past Medical History**	
**Asthma**	20% (3/15)
**Fibroids**	6.67% (1/15)
**Myopia**	20% (3/15)
**Hypertension**	13.33% (2/15)
**GERD**	20% (3/15)
**Hyperopia**	13.33% (2/15)
**Arthritis**	6.67% (1/15)
**Hypothyroidism**	13.33% (2/15)
**Cataract**	6.67% (1/15)
**Osteopenia**	6.67% (1/15)
**Breast cancer (in remission)**	6.67% (1/15)
**Osteoarthritis**	6.67% (1/15)

**Allergies**	
**Seasonal**	6.67% (1/15)
**Dust**	6.67% (1/15)
**Sulfa**	13.33% (2/15)
**Ceclor**	6.67% (1/15)
**Dairy**	6.67% (1/15)
**Unknown medications**	6.67% (1/15)
**Current Medications**	
**Metoprolol**	6.67% (1/15)
**Inhaler**	6.67% (1/15)
**Natural D-Hist**	6.67% (1/15)
**Vitamin C**	6.67% (1/15)
**Aspirin**	6.67% (1/15)
**Losartin**	6.67% (1/15)
**Protonix**	6.67% (1/15)
**Amlodipine**	6.67% (1/15)
**Meprazole**	6.67% (1/15)
**Naprosyn**	6.67% (1/15)
**Vitamin B**	6.67% (1/15)
**Multivitamin**	6.67% (1/15)
**Fish Oil**	6.67% (1/15)
**Zinc**	6.67% (1/15)
**Magnesium**	6.67% (1/15)
**Tumeric**	6.67% (1/15)
**Atenolol**	6.67% (1/15)
**Levothryoxine**	6.67% (1/15)
**Pantoprazole**	6.67% (1/15)
**Lisinopril**	6.67% (1/15)
**Vitamin D3**	6.67% (1/15)
**Lifestyle Habits**	
**Tobacco Use (used to smoke)**	40% (6/15)
**Light Caffeine Use**	6.67% (1/15)
**Heavy Caffeine Use**	66.67% (10/15)
**Light Alcohol Use**	26.67% (4/15)
**Moderate Alcohol Use**	26.67% (4/15)
**Recreational Drug Use**	6.67% (1/15)
**Marijuana Use (Inhalation)**	33.33% (5/15)

Supplementary Table 3. SARS-CoV-2 convalescent ex vivo and cultured average IFNγ ELISpot forming cells per million PBMCs over background stratified by mild and moderate disease

EX VIVO

	MILD COHORT														MODERATE COHORT							
**Donor ID**	CV-002	CV-003	CV-004	CV-006	CV-008	CV-009	CV-011	CV-012	CV-013	CV-015	CV-018	Group Average	Group Standard Deviation	Frequency of Positivity in Group	CV-005	CV-014	CV-016	CV-017	Group Average	Group Standard Deviation	Frequency of Positivity in Group	SOURCE ANTIGEN
**Age**	39	57	53	39	23	58	26	52	41	65	35				60	42	29	53				
**Sex**	Female	Female	Female	Male	Male	Male	Male	Female	Male	Female	Female				Male	Male	Female	Female				
**HLA Type**	01:02/11:04	15:02/15:02	15:01/15:01	09:01/09:01	07:01/11:04	01:01/07:01	11:04/11:04	14:01/15:02	01:02/14:01	01:01/15:01	08:04/15:03				11:04/15:01	09:01/09:01	11:01/15:01	04:03/14:01				
**PEPTIDE ID**																						
**1**	2.7	0.0	5.3	25.3	1.0	27.7	1.0	0.0	16.0	0.0	0.0	7.2	10.6	18%	4.0	0.0	6.7	46.0	14.2	21.4	25%	ENVELOPE
**2**	4.0	2.7	1.3	4.0	14.3	39.7	3.7	1.3	32.0	0.0	0.0	9.4	13.8	18%	110.7	4.0	20.0	83.3	54.5	50.7	50%	MEMBRANE
**3**	5.3	0.0	2.7	0.0	3.7	6.3	0.0	0.0	8.0	0.0	0.0	2.4	3.0	0%	21.3	1.3	1.3	4.7	7.2	9.6	0%	
**4**	9.3	0.0	1.3	90.7	6.3	31.7	0.0	0.0	14.7	0.0	0.0	14.0	27.2	18%	160.0	2.7	0.0	18.0	45.2	77.0	25%	
**5**	8.0	0.0	0.0	4.0	19.7	43.7	11.7	0.0	24.0	0.0	2.3	10.3	13.9	9%	50.7	1.3	2.7	76.7	32.8	37.2	50%	
**6**	13.3	1.3	0.0	28.0	38.3	74.3	14.3	0.0	21.3	0.0	31.7	20.2	22.6	36%	121.3	6.7	10.7	31.3	42.5	53.7	50%	
**7**	12.0	4.0	2.7	25.3	53.0	99.7	5.0	1.3	37.3	0.0	23.7	24.0	30.3	36%	136.0	4.0	29.3	82.0	62.8	58.6	75%	
**8**	16.0	6.7	2.7	29.3	14.3	237.0	0.0	1.3	8.0	1.3	10.3	29.7	69.3	18%	109.3	16.0	6.7	12.7	36.2	48.9	25%	
**9**	10.7	0.0	0.0	1.3	14.3	62.3	1.0	1.3	32.0	0.0	1.0	11.3	19.6	18%	58.7	10.7	9.3	39.3	29.5	23.9	50%	
**10**	1.3	0.0	1.3	32.0	11.7	13.0	0.0	0.0	8.0	0.0	0.0	6.1	9.9	9%	17.3	13.3	4.0	27.3	15.5	9.7	25%	SPIKE – S1
**11**	5.3	4.0	0.0	18.7	27.7	181.0	1.0	5.3	26.7	10.7	17.0	27.0	52.0	27%	110.7	10.7	9.3	71.3	50.5	49.4	50%	
**12**	0.0	0.0	1.3	0.0	1.0	9.0	0.0	1.3	1.3	0.0	0.0	1.3	2.6	0%	5.3	5.3	2.7	8.7	5.5	2.5	0%	
**13**	2.7	0.0	0.0	2.7	3.7	10.3	0.0	1.3	28.0	0.0	0.0	4.4	8.4	9%	70.7	9.3	2.7	79.3	40.5	40.1	50%	
**14**	0.0	1.3	0.0	2.7	13.0	18.3	0.0	1.3	4.0	1.3	1.0	3.9	6.1	0%	16.0	2.7	6.7	14.0	9.8	6.2	0%	
**15**	1.3	4.0	0.0	2.7	5.0	58.3	0.0	0.0	8.0	1.3	5.0	7.8	17.0	9%	36.0	5.3	4.0	20.7	16.5	15.0	25%	
**16**	9.3	0.0	0.0	0.0	11.7	0.0	0.0	0.0	17.3	0.0	1.0	3.6	6.2	0%	100.0	0.0	6.7	79.3	46.5	50.6	50%	
**17**	0.0	0.0	4.0	12.0	7.7	2.3	0.0	1.3	1.3	1.3	0.0	2.7	3.8	0%	9.3	0.0	4.0	0.7	3.5	4.3	0%	
**18**	2.7	0.0	1.3	0.0	3.7	2.3	0.0	0.0	1.3	1.3	2.3	1.4	1.3	0%	17.3	0.0	4.0	7.3	7.2	7.4	0%	
**19**	1.3	0.0	4.0	28.0	21.0	38.3	0.0	0.0	6.7	2.7	0.0	9.3	13.5	18%	26.7	44.0	2.7	14.0	21.8	17.7	50%	
**20**	6.7	0.0	1.3	8.0	9.0	81.0	0.0	0.0	25.3	1.3	0.0	12.1	24.1	18%	77.3	8.0	5.3	76.7	41.8	40.6	50%	
**21**	0.0	0.0	0.0	0.0	7.7	5.0	0.0	1.3	14.7	0.0	0.0	2.6	4.8	0%	10.7	4.0	9.3	10.0	8.5	3.0	0%	
**22**	0.0	6.7	1.3	17.3	17.0	157.0	0.0	2.7	16.0	1.3	1.0	20.0	46.0	9%	60.0	16.0	6.7	32.7	28.8	23.4	50%	
**23**	0.0	0.0	0.0	0.0	1.0	5.0	0.0	0.0	0.0	0.0	2.3	0.8	1.6	0%	0.0	2.7	1.3	7.3	2.8	3.2	0%	
**24**	14.7	0.0	2.7	0.0	17.0	2.3	0.0	0.0	14.7	0.0	1.0	4.8	7.0	0%	57.3	0.0	4.0	47.3	27.2	29.4	50%	SPIKE – S2
**25**	0.0	0.0	0.0	0.0	6.3	1.0	0.0	0.0	8.0	0.0	3.7	1.7	2.9	0%	86.7	1.3	4.0	12.7	26.2	40.6	25%	
**26**	13.3	4.0	0.0	0.0	14.3	31.7	0.0	0.0	33.3	2.7	1.0	9.1	12.7	18%	80.0	0.0	10.7	43.3	33.5	36.1	50%	
**27**	2.7	0.0	0.0	40.0	3.7	5.0	0.0	0.0	12.0	0.0	0.0	5.8	11.9	9%	36.0	6.7	0.0	11.3	13.5	15.7	25%	
**28**	0.0	1.3	0.0	46.7	2.3	1.0	0.0	0.0	6.7	0.0	0.0	5.3	13.9	9%	4.0	17.3	1.3	4.7	6.8	7.1	0%	
**29**	0.0	0.0	0.0	0.0	0.0	0.0	0.0	0.0	0.0	0.0	0.0	0.0	0.0	0%	18.7	0.0	0.0	0.7	4.8	9.2	0%	
**30**	2.7	0.0	1.3	22.7	1.0	0.0	0.0	1.3	1.3	0.0	0.0	2.8	6.7	0%	5.3	0.0	2.7	0.7	2.2	2.4	0%	
**31**	0.0	0.0	0.0	6.7	2.3	10.3	6.3	0.0	6.7	1.3	0.0	3.1	3.7	0%	10.7	21.3	2.7	20.7	13.8	8.9	0%	
**32**	1.3	0.0	1.3	1.3	6.3	3.7	2.3	0.0	0.0	1.3	0.0	1.6	1.9	0%	17.3	1.3	1.3	12.7	8.2	8.1	0%	
**Number of Peptides Identified Per Donor**	0	0	0	9	3	14	0	0	7	0	1	3.1	4.8	---	18	1	1	14	8.5	8.8	---	

CULTURED

	MILD COHORT														MODERATE COHORT							
**Donor ID**	CV-002	CV-003	CV-004	CV-006	CV-008	CV-009	CV-011	CV-012	CV-013	CV-015	CV-018	Group Average	Group Standard Deviation	Frequency of Positivity in Group	CV-005	CV-014	CV-016	CV-017	Group Average	Group Standard Deviation	Frequency of Positivity in Group	SOURCE ANTIGEN
**Age**	39	57	53	39	23	58	26	52	41	65	35				60	42	29	53				
**Sex**	Female	Female	Female	Male	Male	Male	Male	Female	Male	Female	Female				Male	Male	Female	Female				
**HLA Type**	01:02/11:04	15:02/15:02	15:01/15:01	09:01/09:01	07:01/11:04	01:01/07:01	11:04/11:04	14:01/15:02	01:02/14:01	01:01/15:01	08:04/15:03				11:04/15:01	09:01/09:01	11:01/15:01	04:03/14:01				
**PEPTIDE ID**																						
**1**	10.0	0.0	831.7	23.3	0.0	1.7	0.0	0.0	6.7	0.0	40.0	83.0	248.6	18%	3.3	0.0	0.0	13.3	4.2	6.3	0%	ENVELOPE
**2**	53.3	33.3	501.7	56.7	13.3	1.7	3.3	0.0	13.3	3.3	43.3	65.8	146.1	45%	3.3	33.3	0.0	23.3	15.0	16.0	25%	MEMBRANE
**3**	6.7	20.0	48.3	0.0	0.0	1.7	3.3	0.0	13.3	0.0	6.7	9.1	14.5	9%	0.0	0.0	0.0	6.7	1.7	3.3	0%	
**4**	80.0	3.3	145.0	110.0	13.3	1.7	540.0	0.0	36.7	0.0	273.3	109.4	166.1	55%	13.3	53.3	0.0	123.3	47.5	55.4	50%	
**5**	6.7	10.0	138.3	3.3	0.0	1.7	0.0	0.0	0.0	0.0	106.7	24.2	49.2	18%	0.0	6.7	0.0	40.0	11.7	19.1	25%	
**6**	3.3	6.7	11.7	93.3	0.0	5.0	0.0	0.0	13.3	0.0	430.0	51.2	128.5	18%	0.0	13.3	0.0	33.3	11.7	15.8	25%	
**7**	60.0	3.3	11.7	83.3	0.0	11.7	0.0	0.0	16.7	0.0	83.3	24.5	33.8	27%	23.3	36.7	0.0	26.7	21.7	15.5	50%	
**8**	6.7	0.0	8.3	3.3	0.0	5.0	3.3	0.0	6.7	0.0	133.3	15.2	39.3	9%	6.7	16.7	0.0	10.0	8.3	6.9	0%	
**9**	486.7	143.3	90.0	76.7	0.0	30.0	0.0	0.0	80.0	0.0	286.7	108.5	152.1	64%	1276.7	10.0	10.0	10.0	326.7	633.3	25%	
**10**	220.0	6.7	83.3	580.0	6.7	10.0	0.0	0.0	0.0	0.0	210.0	101.5	179.6	36%	786.7	110.0	3.3	3.3	225.8	377.3	50%	SPIKE – S1
**11**	1420.0	223.3	120.0	780.0	0.0	360.0	0.0	3.3	130.0	0.0	1053.3	371.8	492.4	64%	1830.0	66.7	3.3	60.0	490.0	893.8	75%	
**12**	33.3	0.0	610.0	50.0	0.0	6.7	0.0	0.0	0.0	0.0	26.7	66.1	181.2	27%	83.3	0.0	6.7	0.0	22.5	40.7	25%	
**13**	736.7	0.0	483.3	3.3	3.3	0.0	0.0	0.0	23.3	3.3	693.3	177.0	302.2	27%	1693.3	6.7	3.3	36.7	435.0	839.0	50%	
**14**	276.7	3.3	720.0	63.3	0.0	3.3	0.0	0.0	6.7	3.3	186.7	114.8	221.1	36%	406.7	6.7	0.0	0.0	103.3	202.2	25%	
**15**	336.7	53.3	76.7	120.0	0.0	23.3	0.0	0.0	0.0	0.0	353.3	87.6	133.2	45%	1103.3	3.3	0.0	0.0	276.7	551.1	25%	
**16**	1166.7	0.0	476.7	0.0	0.0	0.0	0.0	0.0	56.7	0.0	356.7	187.0	365.3	36%	1493.3	3.3	3.3	50.0	387.5	737.6	50%	
**17**	178.3	3.3	165.0	66.7	0.0	0.0	0.0	0.0	3.3	0.0	130.0	49.7	73.0	36%	13.3	0.0	10.0	10.0	8.3	5.8	0%	
**18**	21.7	40.0	41.7	0.0	1.7	3.3	0.0	0.0	3.3	0.0	23.3	12.3	16.5	18%	10.0	3.3	30.0	13.3	14.2	11.3	25%	
**19**	88.3	56.7	611.7	3206.7	0.0	73.3	0.0	0.0	3.3	0.0	266.7	391.5	951.7	55%	3.3	3603.3	3.3	3.3	903.3	1800.0	25%	
**20**	881.7	26.7	5.0	340.0	1.7	86.7	0.0	0.0	0.0	0.0	413.3	159.5	281.4	45%	36.7	596.7	13.3	10.0	164.2	288.6	50%	
**21**	125.0	6.7	65.0	1196.7	1.7	0.0	3.3	0.0	3.3	0.0	223.3	147.7	355.2	36%	26.7	276.7	50.0	3.3	89.2	126.4	75%	
**22**	915.0	46.7	5.0	773.3	0.0	263.3	0.0	0.0	13.3	0.0	556.7	233.9	348.5	45%	80.0	1100.0	70.0	16.7	316.7	523.0	75%	
**23**	1.7	0.0	0.0	0.0	0.0	0.0	0.0	0.0	0.0	0.0	23.3	2.3	7.0	0%	0.0	10.0	3.3	0.0	3.3	4.7	0%	
**24**	1455.0	0.0	451.7	0.0	1.7	0.0	3.3	0.0	3.3	0.0	250.0	196.8	442.4	27%	23.3	3.3	20.0	10.0	14.2	9.2	0%	SPIKE – S2
**25**	246.7	5.0	8.3	0.0	0.0	10.0	0.0	0.0	0.0	0.0	181.7	41.1	86.9	18%	48.3	0.0	155.0	0.0	50.8	73.1	50%	
**26**	1196.7	31.7	1601.7	3.3	0.0	200.0	3.3	0.0	15.0	0.0	385.0	312.4	558.0	45%	151.7	0.0	278.3	153.3	145.8	113.9	75%	
**27**	60.0	11.7	8.3	0.0	0.0	3.3	0.0	0.0	1.7	0.0	11.7	8.8	17.6	9%	15.0	3.3	21.7	3.3	10.8	9.1	0%	
**28**	66.7	5.0	8.3	0.0	0.0	6.7	0.0	0.0	0.0	0.0	33.3	10.9	20.9	18%	15.0	3.3	28.3	3.3	12.5	11.9	25%	
**29**	20.0	1.7	115.0	0.0	0.0	0.0	3.3	0.0	0.0	0.0	48.3	17.1	35.7	18%	11.7	0.0	8.3	3.3	5.8	5.2	0%	
**30**	3.3	1.7	2121.7	23.3	0.0	6.7	0.0	0.0	0.0	0.0	31.7	198.9	637.8	18%	38.3	0.0	0.0	0.0	9.6	19.2	25%	
**31**	153.3	0.0	348.3	10.0	0.0	3.3	0.0	0.0	21.7	0.0	81.7	56.2	108.3	27%	131.7	96.7	158.3	56.7	110.8	44.1	100%	
**32**	446.7	0.0	28.3	0.0	0.0	0.0	0.0	0.0	1.7	0.0	151.7	57.1	136.9	27%	108.3	3.3	125.0	56.7	73.3	55.0	75%	
**Number of Peptides Identified Per Donor**	23	9	23	15	0	6	1	0	4	0	27	9.8	10.4	---	16	10	8	10	11.0	3.5	---	

Supplementary Table 4. Frequencies of ELISpot assay responses by antigen and culture condition

Source Antigen	Envelope (#1)	Membrane (#2-9)	Spike (#10-32)	S1 (#10-23)	S2 (#24-32)	RBD (#18-22)
**Ex vivo-only**	**3 (20%)**	**20 (17%)**	**14 (4%)**	**8 (4%)**	**6 (4%)**	**4 (5%)**
**Cultured-only**	**2 (13%)**	**25 (21%)**	**94 (27%)**	**62 (29.5%)**	**32 (24%)**	**25 (33%)**
**Both Assays**	**0 (0%)**	**10 (8%)**	**21 (6%)**	**17 (8%)**	**4 (3%)**	**7 (9%)**
**No Recognition**	**10 (67%)**	**65 (54%)**	**216 (63%)**	**123 (58.5%)**	**93 (69%)**	**39 (52%)**
**Freq. of ex vivo positives lost after culture**	100%	67%	40%	32%	60%	36%
**Freq. of "cultured-only" (% of all unique/positive donor-peptide pairs)**	40%	45%	73%	71%	76%	71%
