# Supplementary material for: Highly conserved, non-human-like, and cross-reactive SARS-CoV-2 T cell epitopes for COVID-19 vaccine design and validation
Source: NPJ Vaccines. 2021 May 13;6:71. doi: 10.1038/s41541-021-00331-6 (PMC8119491; doi:10.1038/s41541-021-00331-6)
Supplement: Supplementary file 2 — Reporting Summary [file 41541_2021_331_MOESM2_ESM.pdf]

nature research

Corresponding author(s): Leonard Moise, Anne De Groot

Last updated by author(s): Mar 25, 2021

# Reporting Summary

Nature Research wishes to improve the reproducibility of the work that we publish. This form provides structure for consistency and transparency in reporting. For further information on Nature Research policies, see our Editorial Policies and the Editorial Policy Checklist.

## Statistics

For all statistical analyses, confirm that the following items are present in the figure legend, table legend, main text, or Methods section.

| n/a | Confirmed | |
|---|---|---|
| ☐ | ☒ | The exact sample size (*n*) for each experimental group/condition, given as a discrete number and unit of measurement |
| ☒ | ☐ | A statement on whether measurements were taken from distinct samples or whether the same sample was measured repeatedly |
| ☐ | ☒ | The statistical test(s) used AND whether they are one- or two-sided<br>*Only common tests should be described solely by name; describe more complex techniques in the Methods section.* |
| ☒ | ☐ | A description of all covariates tested |
| ☒ | ☐ | A description of any assumptions or corrections, such as tests of normality and adjustment for multiple comparisons |
| ☐ | ☒ | A full description of the statistical parameters including central tendency (e.g. means) or other basic estimates (e.g. regression coefficient) AND variation (e.g. standard deviation) or associated estimates of uncertainty (e.g. confidence intervals) |
| ☐ | ☒ | For null hypothesis testing, the test statistic (e.g. *F*, *t*, *r*) with confidence intervals, effect sizes, degrees of freedom and *P* value noted<br>*Give P values as exact values whenever suitable.* |
| ☒ | ☐ | For Bayesian analysis, information on the choice of priors and Markov chain Monte Carlo settings |
| ☒ | ☐ | For hierarchical and complex designs, identification of the appropriate level for tests and full reporting of outcomes |
| ☐ | ☒ | Estimates of effect sizes (e.g. Cohen's *d*, Pearson's *r*), indicating how they were calculated |

*Our web collection on statistics for biologists contains articles on many of the points above.*

## Software and code

Policy information about availability of computer code

| | |
|---|---|
| Data collection | Epitope predictions were made with EpiMatrix 1.3 (EpiVax, Providence, RI). Cross-conservation epitope prediction was done with JanusMatrix (EpiVax, Providence, RI). Fluorospot data were collected with iSpot Spectrum 7.0, build 14790 (AID,Strassberg, Germany). Flow cytometry data were collected with Attune NxT Software 4.2.0 (ThermoFisher, Waltham, MA). |
| Data analysis | Flow cytometry data were analyzed using FlowJo 10.6.2 (BD, Ashland, OR). Statistical analysis was performed using Prism 9.0.0 (GraphPad Software, San Diego, CA). |

For manuscripts utilizing custom algorithms or software that are central to the research but not yet described in published literature, software must be made available to editors and reviewers. We strongly encourage code deposition in a community repository (e.g. GitHub). See the Nature Research guidelines for submitting code & software for further information.

## Data

Policy information about availability of data

All manuscripts must include a data availability statement. This statement should provide the following information, where applicable:

- Accession codes, unique identifiers, or web links for publicly available datasets
- A list of figures that have associated raw data
- A description of any restrictions on data availability

Data generated or analysed during this study that are critical to the reported findings are included in this published article and its supplementary information files. Additional supporting data are available from the corresponding author on reasonable request.

# Field-specific reporting

Please select the one below that is the best fit for your research. If you are not sure, read the appropriate sections before making your selection.

☒ Life sciences ☐ Behavioural & social sciences ☐ Ecological, evolutionary & environmental sciences

For a reference copy of the document with all sections, see nature.com/documents/nr-reporting-summary-flat.pdf

# Life sciences study design

All studies must disclose on these points even when the disclosure is negative.

| | |
|---|---|
| Sample size | The sample size was based on how many individuals could be recruited between May and December 2020. |
| Data exclusions | One mouse in the vaccine group that received 5 ug/peptide was excluded following splenocyte isolation due to insufficient recovery and poor viability for reasons thought to be unrelated to vaccination. |
| Replication | All experiments were completed with technical replicates; triplicates in Fluorospot assays and flow cytometry assays. A subset of healthy and convalescent donor samples were assayed by Fluorospot assay twice and experimental findings were replicated. |
| Randomization | This is not relevant as this is an observational study. |
| Blinding | This is not relevant as this is an observational study. |

# Reporting for specific materials, systems and methods

We require information from authors about some types of materials, experimental systems and methods used in many studies. Here, indicate whether each material, system or method listed is relevant to your study. If you are not sure if a list item applies to your research, read the appropriate section before selecting a response.

## Materials & experimental systems

| n/a | Involved in the study | |
|---|---|---|
| ☐ | ☒ | Antibodies |
| ☒ | ☐ | Eukaryotic cell lines |
| ☒ | ☐ | Palaeontology and archaeology |
| ☐ | ☒ | Animals and other organisms |
| ☐ | ☒ | Human research participants |
| ☒ | ☐ | Clinical data |
| ☒ | ☐ | Dual use research of concern |

## Methods

| n/a | Involved in the study | |
|---|---|---|
| ☒ | ☐ | ChIP-seq |
| ☐ | ☒ | Flow cytometry |
| ☒ | ☐ | MRI-based neuroimaging |

## Antibodies

Antibodies used

Flow cytometry:
syrian hamster anti-mouse CD3e-AF700, clone 500A2, BioLegend, 152316
rat anti-mouse CD4-APC/Fire750, clone GK1.5, BioLegend, 100460
rat anti-mouse CD8a-FITC, clone 53-6.7, BioLegend, 100702
rat anti-mouse CD62L-APC, clone MEL-14, BioLegend, 104428
rat anti-mouse CD44-eFLuor506, clone IM7, ThermoFisher, 14-0441-82
rat anti-mouse IFNg-BV605, clone XMG1.2, BioLegend, 505840
rat anti-mouse IL-4- PerCP/Cy5.5, clone 11B11, BioLegend 504124
rat anti-mouse IL-5-PE, clone TRFK5, BioLegend 504304

Human Fluorospot assay:
mouse anti-human IFNg, clone 1-D1K, Mabtech 3420-3-250 (in human IFNg Fluorospot kit, FSP-01A-10)
mouse anti-human IFNg, clone 7-B6-1-BAM, Mabtech in FSP-01A-10
anti-BAM-490, Mabtech in FSP-01A-10

Mouse Fluorospot assay:
rat anti-mouse IFNg, clone AN18, Mabtech 3321-3-250 (in mouse IFNg/IL-4 Fluorospot kit, FSP-4142-10)
rat anti-mouse IL-4, clone 11B11, Mabtech 3311-3-250 in FSP-4142-10
anti-SA-550, Mabtech in FSP-4142-10
rat anti-mouse IFNg, clone R4-6A2-BAM, Mabtech in FSP-4142-10
rat anti-mouse IL-4, clone BVD6-24G2, Mabtech 3311-6-250 in FSP-4142-10

Validation

BioLegend antibody quality control and validation statements are found at: https://www.biolegend.com/en-us/quality-control, https://www.biolegend.com/en-us/reproducibility

ThermoFisher eBioscience CD44-eFLuor506 antibody validation data available at https://www.thermofisher.com/order/genome-database/dataSheetPdf?producttype=antibody&productsubtype=antibody_primary&productId=14-0441-82&version=137

## Animals and other organisms

Policy information about studies involving animals; ARRIVE guidelines recommended for reporting animal research

| | |
|---|---|
| Laboratory animals | Mus musculus, H2dlAb1-Ea Tg(DRA1*0103,DRB1*0301), female, 6-8 weeks at start of study |
| Wild animals | The study did not involve wild animals. |
| Field-collected samples | The study did not involve samples collected from the field. |
| Ethics oversight | Animal research protocols for mouse studies were reviewed and approved by the Absorption Systems Inc. Institutional Animal Care and Use Committee. |

Note that full information on the approval of the study protocol must also be provided in the manuscript.

## Human research participants

Policy information about studies involving human research participants

| | |
|---|---|
| Population characteristics | COVID-19 convalescents with PCR-confirmed SARS-CoV-2 infection (N=15) over March-June 2020 were recruited between 30 and 180 days after their most recent positive test and a minimum of 14 days after symptoms resolved. Donors exhibited a wide range of COVID-19 symptoms and experienced either mild or moderate disease according to WHO criteria. Blood draws from convalescents ranged from approximately one to six months from diagnosis. Healthy individuals (N=10) provided cell samples from February 2016 up to November 2019 and had no opportunity for SARS-CoV-2 exposure. Both cohorts contain a balanced proportion of females and males and similar average age and age range. 60% of convalescent donors are from racial and ethnic minorities. No ethnicity information is available for the healthy cohort. |
| Recruitment | COVID-19 convalescent patients were recruited by Sanguine Biosciences, a clinical services group that identified, consented and enrolled participants. Inclusion criteria included subjects (i) willing and able to provide written informed consent, (ii) aged 18-80 years of age, both male or female, and (iii) PCR-confirmed COVID-19 diagnosis (recovered) with date of diagnosis a minimum of 30 days from blood collection. Exclusion criteria included subjects who (i) were pregnant or nursing, (ii) had a known history of HIV, hepatitis or other infectious diseases, (iii) had autoimmune diseases, (iv) were members of vulnerable patient population (prisoners, mentally impaired), (v) had medical conditions impacting their ability to donate blood (i.e. anemia, acute illness) (vi) had received immunosuppressive therapy or steroids within the last 6 months, (vii) had received an investigational product in the last 30 days, (viii) had experienced excess blood loss including blood donation defined as 250 mL in the last month or 500 mL in the last two months, or (ix) had a positive COVID-19 PCR test, but were asymptomatic.<br><br>Deidentified samples from healthy unexposed donors were obtained from leukocyte reduction filters from the Rhode Island Blood Center for unrelated studies prior to the SARS-CoV-2 outbreak in December 2019 (Date of samples: February 2016 – November 2019). |
| Ethics oversight | Ethical & Independent Review Services, Independence, MO |

Note that full information on the approval of the study protocol must also be provided in the manuscript.

## Flow Cytometry

### Plots

Confirm that:

☒ The axis labels state the marker and fluorochrome used (e.g. CD4-FITC).

☒ The axis scales are clearly visible. Include numbers along axes only for bottom left plot of group (a 'group' is an analysis of identical markers).

☒ All plots are contour plots with outliers or pseudocolor plots.

☒ A numerical value for number of cells or percentage (with statistics) is provided.

### Methodology

| | |
|---|---|
| Sample preparation | Spleens were obtained from immunized mice. Erythrocyte-depleted, single cell splenocyte suspensions were prepared following mechanical spleen disruption. |
| Instrument | Attune NxT AFC2 flow cytometer (ThermoFisher/Invitrogen) |

| | |
|---|---|
| Software | Flow cytometry data were collected with Attune NxT Software 4.2.0 (ThermoFisher, Waltham, MA). Flow cytometry data were analyzed using FlowJo 10.6.2 (BD, Ashland, OR). |
| Cell population abundance | The study involved no cell sorting for preparation of purified populations. |
| Gating strategy | Cells were first gated for lymphocytes in a SSC-A (y-axis) versus FSC-A (x-axis) plot. Single cells were identified by plotting FSC-A (y-axis) versus FSC-H (x-axis). Live cells were then gated in a FSC-A (y-axis) versus Live/Dead fixable stain 450 (x-axis). CD4 T cells were then identified in a CD3e-AF700 (y-axis) versus CD4-APC/Fire750 (x-axis) plot. CD8 T cells were then identified in a CD3e-AF700 (y-axis) versus CD8a-FITC (x-axis) plot. Next, CD44+ memory CD4 T cells and CD44+ memory CD8 T cells were defined on CD44-eFLuor506 (y-axis) versus CD62L-APC (x-axis) plots. Cytokine expression was identified in CD44+ CD4 T cells or CD44+ CD8 T cells on plots of CD44-eFLuor506 (y-axis) versus IFNg-BV605 (x-axis) or IL-4-PerCP/Cy5.5 (x-axis) or IL-5-PE (x-axis). |

☒ Tick this box to confirm that a figure exemplifying the gating strategy is provided in the Supplementary Information.
